# Fingerprint Profile Analysis of *Eupolyphaga steleophaga* Polypeptide Based on UHPLC-MS and Its Application

**DOI:** 10.3390/ph18020166

**Published:** 2025-01-26

**Authors:** Xin Lai, Hongwei Song, Guangli Yan, Junling Ren, Xijun Wang

**Affiliations:** State Key Laboratory of Integration and Innovation of Classic Formula and Modern Chinese Medicine, Heilongjiang University of Chinese Medicine, Harbin 150040, China; 18834187534@163.com (X.L.); magnisong@sina.com (H.S.); junlingren@126.com (J.R.); xijunw@sina.com (X.W.)

**Keywords:** *Eupolyphaga steleophaga* polypeptide, fingerprint profile, quality identification, ultrahigh-performance liquid chromatography-mass spectrometry (UHPLC-MS)

## Abstract

**Background and Objectives:** As a medicinal and food homologous substance, *Eupolyphaga steleophaga* is renowned for its potential health benefits, including anti-tumor effects, immune system support, and anti-inflammatory properties. *Eupolyphaga steleophaga* polypeptides have demonstrated significant biological activity, including the regulation of coagulation and lipid metabolism. However, the peptide composition of *Eupolyphaga steleophaga* requires further clarification to facilitate quality control improvements and a deeper investigation into its pharmacological effects. Therefore, this study aimed to simulate the digestive absorption process of *Eupolyphaga steleophaga* following oral administration and identify its enzymatic components to enhance quality control. **Methods**: The digestive absorption process was simulated using artificial gastric fluid and pepsin. A fingerprinting method based on ultrahigh-performance liquid chromatography-mass spectrometry (UHPLC-MS)(Acquire UPLC-Synapt G2-Si HDMS, Waters Corporation, Milford, MA, USA) was developed to identify 63 enzymatic components. The enzymolysis polypeptide fingerprint detection method was used to analyze 10 batches of *Eupolyphaga steleophaga* sourced from Harbin No. 4 Traditional Chinese Medicine Factory. Chromatographic collection was performed using an ACQUITY UPLC BHE C18 column. Gradient elution was carried out using a mixture of 0.1% formic acid with acetonitrile and 0.1% formic acid with water, with an average flow rate of 0.3 mL/min, a column temperature of 40 °C, and an injection volume of 2 μL. The mass spectrometry (MS) conditions were set as follows: the ion source was operated in positive electrospray ionization (ESI+) mode, with a capillary voltage of 2.8 kV and a sampling cone voltage of 40 V. The ion-source temperature was maintained at 110 °C, while the desolvation temperature was set to 400 °C. The cone gas flow rate was 50 L/h, and the desolvation gas flow rate was 800 L/h. The range for the collection of mass-to-charge ratios (*m*/*z*) was between 50 and 1200. **Results:** The UHPLC-MS method demonstrated high accuracy, repeatability, and stability, successfully identifying 63 enzymatic components of *Eupolyphaga steleophaga*. Furthermore, polypeptide markers for 63 selected components were identified in all 10 batches of *Eupolyphaga steleophaga* medicinal materials. This approach was validated by including numerical values such as retention times and peak areas, confirming its reliability for quality control enhancement. **Conclusions:** This novel UHPLC-MS approach serves as a powerful tool for advancing quality control strategies in veterinary medicine, particularly for animal-derived medicines. It lays a solid foundation for subsequent pharmacological studies of *Eupolyphaga steleophaga* polypeptides, offering a more reliable means to explore their biological activities and therapeutic potential.

## 1. Introduction

*Eupolyphaga steleophaga*, the dry body of the female insect *Eupolyphaga sinensis Walker*, or *Steleophaga plancyi (Boleny)*, is rich in protein, vitamins, essential amino acids, minerals, and fatty acids, and has long been used as a food source. Concurrently, it has been used as medicine in Traditional East Asian practices [1,2,3], as it has the functions of removing blood stasis, promoting blood circulation, dispersing lumps, and promoting bone and muscle healing [4,5]. Modern studies have shown that bioactive components such as polypeptides, amino acids, and flavonoids in *Eupolyphaga steleophaga* possess anti-angiogenic properties. In particular, the polypeptides in *Eupolyphaga steleophaga* are closely associated with anticoagulant effects and the regulation of blood lipids [4,6,7]. Polypeptides are functional components that are usually extracted from animal and plant proteins. Traditional Chinese medicine polypeptides are peptides consisting of 2 or more amino acids with relative molecular masses between small molecules and proteins that express the effects of traditional Chinese medicine [8]. The process of polypeptide generation begins with the transcription of DNA into messenger RNA (mRNA), which is then translated by ribosomes in the cytoplasm into a nascent polypeptide chain. This chain may undergo further modifications to produce a fully functional polypeptide. In many cases, polypeptides are initially synthesized as part of larger precursor proteins, which must be processed to become biologically active. These precursor proteins are often inactive or require cleavage at specific sites to release the active polypeptide. For example, insulin is synthesized as a precursor protein known as preproinsulin, which undergoes cleavage to remove the signal peptide, followed by further cleavage to generate the active hormone, insulin [9]. Other polypeptides are generated from inactive enzyme precursors, known as zymogens, which are activated by proteolytic cleavage. For example, trypsinogen is an inactive precursor of trypsin, a digestive enzyme, and it is activated by the enzyme enteropeptidase in the small intestine [10]. When polypeptides are part of a parent protein, they are inactive. These peptides exist in an inactive form within the long chains of proteins, and their activity is released upon hydrolysis by specific proteases [11]. Once released, these bioactive peptides can be absorbed intact into the bloodstream, where they participate in various physiological processes such as feeding, digestion, metabolism, immune system regulation, and the modulation of numerous biological functions [12,13]. In recent years, numerous bioactive peptides with physiological activity have been discovered and developed. The components such as EPS, A4, etc., have antioxidant effects [11], EPS72 has antitumor effects [14], and P9 has anticoagulant effects [6].

Current research on the chemical composition of *Eupolyphaga steleophaga* primarily focuses on small molecules like amino acids, lipids, polysaccharides, and alkaloids. However, there has been limited research on the analysis of polypeptides, which are important active components. It is essential to conduct fingerprinting analysis of *Eupolyphaga steleophaga* polypeptides, which not only aids in the quality control of the species but also plays a crucial role in furthering the understanding of its pharmacological mechanisms.

Despite the promising bioactivity of its peptides, few studies have comprehensively explored the complex polypeptide profiles of *Eupolyphaga steleophaga*. Various advanced proteomics techniques, including two-dimensional gel electrophoresis (2-DE), multidimensional liquid chromatography (MDLC), matrix-assisted laser desorption/ionization time-of-flight mass spectrometry (MALDI-TOF-MS), and isobaric tags for relative and absolute quantification (iTRAQ), have been employed to analyze traditional Chinese medicines derived from animal sources [15]. Recent advancements in analytical technologies, particularly UHPLC-MS, have revolutionized the profiling of complex biological samples. This technique allows for the high-throughput and detailed analysis of peptides, which is crucial for the fingerprinting and characterization of the chemical profiles in *Eupolyphaga* polypeptides. UHPLC-MS was used to obtain fingerprint profiles of *Eupolyphaga steleophaga* polypeptides, revealing a complex mixture of polypeptides with diverse biological functions. The method highlighted variations in peptide profiles due to factors like age and environment and suggested potential therapeutic polypeptides for further research and drug development.

This study employed UHPLC-MS to analyze the composition of polypeptides in *Eupolyphaga steleophaga*. Polypeptides were extracted from the hydrolysate of artificial gastric fluid mixed with pepsin, followed by their identification and the establishment of a fingerprint profile. This research not only offers a method to improve the quality control of *Eupolyphaga steleophaga* but also provides a foundation for future investigations into the pharmacological mechanisms of its peptides.

## 2. Results

### 2.1. Method Validation of Eupolyphaga steleophaga Polypeptide Fingerprint

#### 2.1.1. Precision Investigation

The test solution containing enzymolysis polypeptides from *Eupolyphaga steleophaga* was prepared following the procedure outlined in the methods section. A single sample was subjected to six sequential injections, and the resulting total ion chromatogram was obtained using UHPLC-MS. Characteristic ion peaks corresponding to the enzymolysis polypeptides of *Eupolyphaga steleophaga* were subsequently identified and extracted from this chromatogram. The characteristic chromatogram, derived from the six injections, is presented in Figure S1. The Relative Standard Deviation (RSD) of retention times for each identified ion was found to be less than 0.3%, while the RSD for the area of each peak was determined to be below 10%, as shown in Tables S2 and S3. These results confirm the high precision and reliability of the analytical method employed.

#### 2.1.2. Repeatability Investigation

Following the procedure outlined in the methods section, six test solutions containing enzymolysis polypeptides from *Eupolyphaga steleophaga* were prepared. Each sample was analyzed three times using UHPLC–MS to obtain the total ion chromatogram. From these chromatograms, characteristic ion peaks corresponding to the enzymolysis polypeptides were identified and extracted, leading to distinct chromatographic profiles for each test solution, as shown in Figure S2. The RSD of the retention times for the characteristic peaks was found to be less than 0.3%, while the RSD of the peak areas was determined to be under 10%, as summarized in Tables S4 and S5. These results indicate that the preparation of the *Eupolyphaga steleophaga* enzymolysis polypeptide test solution exhibited commendable repeatability.

#### 2.1.3. Stability Investigation

The test solution of *Eupolyphaga steleophaga* enzymolysis polypeptides was prepared as described in the methods sections and subsequently injected at intervals of 0, 2, 4, 6, 8, 10, and 12 h. Total ion chromatograms were obtained at each time point using UHPLC-MS. From these chromatograms, characteristic ion peaks corresponding to the enzymolysis polypeptides were identified and extracted, resulting in distinct chromatographic profiles at different time intervals, as illustrated in Figure S3. The RSD of the retention times for each characteristic peak was determined to be below 0.3%, while the RSD of the peak areas remained under 10%, as presented in Tables S6 and S7. These results demonstrate that the test solution of *Eupolyphaga steleophaga* enzymolysis polypeptides remained stable over a duration of 12 h.

### 2.2. UHPLC-MS Detection of Eupolyphaga steleophaga Polypeptide Fingerprint

**Figure 1:**
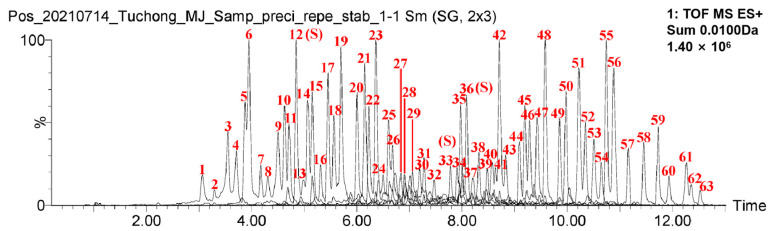
Ultrahigh-performance liquid chromatography-mass spectrometry (UHPLC-MS) fingerprint of *Eupolyphaga steleophaga* enzymolysis polypeptides.

Based on the integrity and characteristic features of the fingerprint profile, it is possible to evaluate and control the quality of traditional Chinese medicine from the perspective of “whole components” even when the active ingredients are not fully identified. Both the U.S. Food and Drug Administration (FDA) and the European Medicines Agency (EMA) have included fingerprint profiles as a method for quality control of herbal mixtures in their guidelines for plant-based medicines. However, there are relatively few reports on the fingerprint profile of *Eupolyphaga steleophaga*. The experimental methodology previously described was employed to successfully prepare a test solution containing enzymolysis polypeptides derived from *Eupolyphaga steleophaga*. The base peak ion chromatogram was obtained using UHPLC-MS, as shown in Figure S4. Following the established data processing protocol, a total of 63 characteristic ions corresponding to the enzymolysis polypeptides were identified and screened. The mass spectra are presented in Figure S5, with relevant details regarding the polypeptide ions provided in Table S1. Using the mass spectrometric data of the identified *Eupolyphaga steleophaga* polypeptide ions, the characteristic ion chromatographic peaks were extracted to generate the UHPLC-MS characteristic spectrum of the polypeptides, as illustrated in Figure 1 below. After analyzing the obtained chromatogram, we have selected the chromatographic peaks with high precision, good repeatability, and stability as the main fingerprints for *Eupolyphaga steleophaga* (No. 12, 33, 36).

### 2.3. Application

*Eupolyphaga steleophaga* is recognized for its medicinal properties as outlined in the Chinese Pharmacopeia, which identifies two primary applications for this species [4]. The earliest documentation of *Eupolyphaga steleophaga* can be traced back to Shennong’s Classic of Materia Medica. The Annotated Catalog of Famous Physicians notes its habitat, stating: “It thrives in marshy and sandy regions of the He Dong area, as well as in moist environments adjacent to residential structures” [16]. The Compendium of Materia Medica characterizes it as “flat and resembling a tortoise, hence referred to as the earth tortoise; it possesses a shell, is incapable of flight, and emits a subtle odor. It is also found in contemporary households” [17]. Furthermore, the Newly Revised Materia Medica describes this organism as flourishing in soil beneath mouse burrows and the foundations of houses, resembling a woodlouse, with larger specimens measuring approximately one inch. Its morphology is likened to that of a tortoise, although it lacks a shell and features scales [18]. These descriptions and illustrations from classical texts align with the current understanding of the medicinal *Eupolyphaga steleophaga* species utilized today. Zhang Ye [19] provided a comprehensive overview of the distinguishing characteristics necessary for the identification of Polyphaga, Phlyphaga plancyi, Opisthoplatia orientalis Burm, and Cybister tripunctatus orientalis Gschew in the article “Identification of *Eupolyphaga steleophaga* and their adulterants”. This study employed UHPLC–MS technology to establish the polypeptide fingerprint of *Eupolyphaga steleophaga* for the first time, thereby offering innovative approaches and methodologies for the quality assessment of this species.

The enzymolysis polypeptide fingerprint detection method for *Eupolyphaga steleophaga*, as detailed in the methods section, was utilized to analyze ten batches of *Eupolyphaga steleophaga* sourced from Harbin No. 4 Traditional Chinese Medicine Factory. The polypeptide fingerprints for the analyzed batches are presented in Figure S6. Retention times and peak areas of the shared peaks across all ten batches were recorded, and the corresponding RSD for these parameters were subsequently calculated. The results, summarized in Tables S8 and S9, demonstrate that the RSD for both retention time and peak area of each common peak remained below 0.35% and 16%, respectively. These values confirm that the fingerprinting data meet the established standards for consistency and reliability. These results demonstrate consistency among the common peaks across the ten batches. Furthermore, polypeptide markers for 63 selected components were identified in all ten batches of *Eupolyphaga steleophaga* medicinal materials. This fingerprint profile appears to provide an effective characterization of the macromolecular proteins found in *Eupolyphaga steleophaga*, indicating its potential as a dependable tool for the quality control of the material.

## 3. Discussion

This study aimed to develop a reliable method for analyzing the fingerprint profile of *Eupolyphaga steleophaga* polypeptides using UHPLC-MS. The results showed that UHPLC-MS is an effective tool for analyzing the complex peptide profiles of *Eupolyphaga steleophaga*, and the method demonstrates high precision, repeatability, and stability. After analyzing the obtained chromatogram, the chromatographic peaks with high precision, repeatability, and stability are selected as the main fingerprint profile of *Eupolyphaga steleophaga*. We also employed UHPLC-MS technology to establish the polypeptide fingerprint of *Eupolyphaga steleophaga*, thereby offering innovative approaches and methodologies for the quality assessment of this species. It revealed distinct fingerprint patterns that can be used for quality control and potential pharmacological studies.

A widely acknowledged consensus regarding the influence of lifestyle on human health highlights the essential role of diet in shaping health outcomes. Animal-derived proteins are acknowledged as a significant source of amino acids during digestion, attributed to their advantageous nutritional characteristics [20]. However, both digestive mechanisms and industrial food processing can liberate bioactive peptides from these parent proteins, which may exert diverse biological effects [21]. These bioactive peptides can be produced through hydrolysis facilitated by digestive enzymes, endogenous enzymes present in the raw materials, or microbial enzymes introduced during food processing. Due to their capacity to affect a broad spectrum of physiological responses in living organisms, animal-derived food proteins have emerged as a prominent source of bioactive peptides, garnering considerable attention in contemporary research [22]. Currently, scholars are concentrating on investigating hydrolysis techniques for food proteins to identify specific peptides, as certain peptides have exhibited bioactive properties and potential health benefits [23,24]. Animals serve as a natural reservoir of bioactive peptides, which offer numerous advantages, including high biological activity, target specificity, selectivity, and minimal adverse effects, thereby becoming a focal point of research and application [25,26]. Research has shown that macromolecular compounds from *Eupolyphaga steleophaga* exhibit significant pharmacological activities, such as antithrombotic, anticancer, and anti-inflammatory effects.

Beyond their nutritional value, bioactive peptides and other bioactive compounds are integral to the management of chronic diseases and the enhancement of overall health [27]. Based on these attributes and functions, numerous animals are utilized as sources of health foods and pharmaceuticals aimed at the prevention and treatment of various ailments. Moreover, advancements in technology have led to the increasing application of chemical analysis methods, including spectroscopy and chromatography, for peptide detection, which is vital for the discovery of bioactive peptides [28,29,30].

*Eupolyphaga steleophaga* polypeptide composition and pharmacological effects have been extensively investigated and acknowledged within the academic community. Various active protein peptides have been extracted from *Eupolyphaga steleophaga* [11,14]. Bioactive peptides, particularly those derived from both plant and animal sources, have been reported to exhibit lipid-lowering activities [31]; however, their underlying mechanisms of action remain inadequately understood. Research has confirmed that the active peptide AR-9 from *Eupolyphaga steleophaga* can mitigate lipid and hepatic lipid accumulation in hyperlipidemic rats by restoring gut microbiota and its metabolites, thereby ameliorating hyperlipidemia and reducing levels of triglycerides, total cholesterol, and low-density lipoprotein, ultimately achieving a lipid-lowering effect [32,33,34]. One study demonstrated the application of *Eupolyphaga steleophaga* in treating a liver cancer cell line (H22), revealing its capacity to inhibit cancer cell proliferation by inducing apoptosis, as evidenced by increased expression of caspase-3 and Bax, along with an elevated Bax/Bcl-2 protein ratio [35].

The existing quality standards for *Eupolyphaga steleophaga* and its associated pharmacological effects reveal considerable inconsistencies, failing to accurately represent the bioactivity of the peptides present. Animal-derived medicines constitute a distinctive aspect of traditional Chinese medicine, noted for their efficacy; however, their pharmacological components remain inadequately understood. Numerous studies indicate that animal-derived peptides are the primary contributors to the therapeutic effects of these medicinal products. However, existing methods for quality control and product evaluation fail to adequately capture their therapeutic efficacy, which limits the progress and development of such treatments. At present, the quality control measures for animal-derived medicines are limited and ineffective, failing to genuinely represent the biological activities of the bioactive peptides they encompass. This situation underscores the significant challenges and pressing issues confronting the field of veterinary medicine [11,36]. To achieve a comprehensive quality evaluation of animal medicines, the issues in the research of their bioactive peptides need to be addressed. This can be approached from the following aspects: (1) strengthening the study of bioactivity for characteristic peptides of animal medicines by utilizing bioinformatics theories and animal models to validate target pathways, and investigating the pharmacological mechanisms of animal peptides while also focusing on underexplored pharmacological effects. (2) In quality control of animal medicines, peptide segments that cannot be accurately identified can be isolated and purified to obtain monomers, followed by sequencing using traditional Edman degradation methods, or their sequences can be inferred through de novo sequencing. Corresponding peptide reference standards can be synthesized and identified using mass spectrometry to confirm their sequences. (3) Research on the protein and peptide components in both raw and processed animal medicines should be conducted to clarify the differences in peptide compositions before and after processing. This can be analyzed in conjunction with changes in pharmacological effects, providing modern research support for traditional theories, such as enhancing efficacy and reducing toxicity through processing, thereby promoting the modernization of animal-based traditional medicines.

Whether in the context of functional foods or pharmaceuticals, quality control is an essential prerequisite for ensuring optimal bioactivity. This study seeks to introduce a novel evaluation method for the quality control of *Eupolyphaga steleophaga* as a medicinal resource, focusing on macromolecular proteins, and aims to enhance research strategies for the quality control of animal-derived medicines.

## 4. Materials and Methods

### 4.1. Materials

The reagents used were acetonitrile and methanol (LC/MS-grade, Thermo Fisher Scientific, Waltham, MA, USA), formic acid (LC/MS-grade; Merck KGaA, Darmstadt, Germany), concentrated hydrochloric acid (analytical grade, Tianjin Kemiou Chemical Reagent Co., Ltd., Tianjin, China), and pepsin (analytical grade, Beijing Solarbio Science & Technology Co., Ltd., Beijing, China).

*Eupolyphaga steleophaga* was provided by Harbin No. 4 Traditional Chinese Medicine Factory Co., Ltd.(Harbin, China). It was identified by Professor Huifeng Sun from Heilongjiang University of Chinese Medicine as the dry body of the female insect Eupolyphaga sinensis Walker, or Steleophaga plancyi (Boleny).

The equipment used were as follows: UPLC (Acquire UPLC, Waters Corporation, Milford, MA, USA), MS (Synapt G2-Si HDMS, Waters Corporation, Milford, MA, USA), electrothermal thermostatic speed-regulating oscillator (SYC-A, Shanghai Xinmiao Medical Device Manufacturing Co., Ltd., Shanghai, China), multi-tube vortex oscillator (VX-II, Beijing Tajin Technology Co., Ltd., Beijing, China), electronic analytical balance (BPI1510-N, Mettler Toledo Instruments, Columbus, OH, USA), and CNC ultrasonic cleaner (KQ-250DB, Kunshan Ultrasonic Instrument Co., Ltd., Kunshan, China).

### 4.2. Methods

#### 4.2.1. Preparation of Simulated Gastric Juice

A volume of 9 mL of concentrated hydrochloric acid (HCl, approximately 10 M, analytical grade) was accurately measured and then diluted with distilled water to reach a final volume of 1000 mL. The prepared solution, with a final concentration of approximately 0.09 M HCl, was transferred into a sealed container and stored in a refrigerator at 4 °C until it was needed for further use [37].

#### 4.2.2. Preparation of *Eupolyphaga steleophaga* Enzymolysis Polypeptide Test Solution

A precise amount of 1.0 g of *Eupolyphaga steleophaga* powder was weighed and transferred into a conical flask with a stopper. To this, 20 mL of artificial gastric juice containing 2% pepsin (*w*/*v*) was added. The flask was then sealed and placed in a constant-temperature shaker set to 40 °C for 1 h to promote the extraction of active components. Afterward, the enzymatic hydrolysate was heated in a constant-temperature water bath at 85 °C for 15 min. The mixture was subsequently cooled to room temperature using running water. The hydrolysate was then centrifuged at 4 °C and 5000 rpm for 10 min. The resulting supernatant was mixed with methanol at a 1:1 ratio and vortexed for 30 s. Following centrifugation at 4 °C and 10,000 rpm for 10 min, 2 mL of the supernatant was carefully collected. Finally, the supernatant was filtered through a 0.22 µm membrane into a sampling bottle for subsequent UHPLC-MS analysis.

#### 4.2.3. Chromatographic Conditions

Chromatographic analysis was performed using an ACQUITY UPLC BHE C18 column (1.7 µm, 100 × 2.1 mm^2^). The mobile phase consisted of solvent A (0.1% formic acid mixed with acetonitrile) and solvent B (0.1% formic acid mixed with water) with the following gradient elution program: 0–5 min, 1–10% A; 5–10 min, 10–30% A; 10–15 min, 30–60% A. The average flow speed was 0.3 mL/min, the ambient temperature of the column was 40 °C, and the amount of fluid infused was 2 μL.

#### 4.2.4. MS Conditions

The mass spectrometry (MS) conditions were set as follows: the ion source was operated in positive electrospray ionization (ESI+) mode, with a capillary voltage of 2.8 kV and a sampling cone voltage of 40 V. The ion-source temperature was maintained at 110 °C, while the desolvation temperature was set to 400 °C. The cone gas flow rate was 50 L/h, and the desolvation gas flow rate was 800 L/h. The range for the collection of mass-to-charge ratios (*m*/*z*) was between 50 and 1200. Data acquisition and subsequent analysis were performed using MassLynx 4.1.

#### 4.2.5. Screening and Identification of *Eupolyphaga steleophaga* Enzymolysis Polypeptides

In this study, enzymatically digested polypeptides from *Eupolyphaga steleophaga* were screened and identified using UHPLC-MS. The process began with the analysis of the total ion chromatogram (TIC) obtained through UHPLC-MS, where ions showing an isotope peak difference of 1/N (N ≥ 2) in their mass-to-charge ratios were selected for further investigation. These polypeptides, synthesized via the dehydration and condensation of amino acids, carry multiple charges in the positive-ion mode of the mass spectrometer.

MassLynx software was employed to filter the ions of interest based on two criteria: first, the presence of nitrogen atoms, and second, the isotope peak difference of 1/N. Ions that met both conditions were classified as target polypeptides. After identifying the relevant ions, the method for extracting the enzymatic digestion polypeptides was optimized. This included refining various extraction parameters such as solvent choice, extraction time, volume of simulated gastric juice, pepsin concentration, temperature, and sampling volume.

The optimization process involved assessing the precision, repeatability, and stability of the extraction method, excluding ions that were unstable or displayed low intensity. Ultimately, the stable, reproducible, and high-intensity ions were identified as enzymatically digested polypeptides from *Eupolyphaga steleophaga*. These selected ions were extracted individually using the ion extraction module in MassLynx software. Finally, a comprehensive UHPLC-MS fingerprint was constructed, representing the enzymolysis polypeptides derived from *Eupolyphaga steleophaga*.

## 5. Conclusions

This research, which focuses on peptides, has successfully developed a fingerprinting profile for the enzymatic peptides of *Eupolyphaga steleophaga* utilizing UHPLC-MS technology for the first time. The method’s accuracy, repeatability, and stability have been rigorously validated. This technique enables the identification of the origin of *Eupolyphaga steleophaga* and has the potential to enhance the quality standards and control measures for both *Eupolyphaga steleophaga* and other Chinese patented medicines that incorporate it and presents a novel approach to improving the quality control processes for *Eupolyphaga steleophaga*. Furthermore, the results may provide valuable insights for the quality control of other medicines or food products derived from animal sources.

## Data Availability

The data that support the findings of this study are available from the corresponding author upon reasonable request.

## Supplementary Materials

The following supporting information can be downloaded at: https://www.mdpi.com/article/10.3390/ph18020166/s1, Figures S1–S3: Method validation of UHPLC-MS fingerprints of *Eupolyphaga steleophaga* enzymolysis polypeptides; Figure S4: UHPLC-MS base peak ion chromatogram of *Eupolyphaga steleophaga* enzymolysis polypeptides; Figure S5: Characteristic mass spectrometry (MS) of *Eupolyphaga steleophaga* enzymolysis polypeptides; Figure S6: Ultrahigh-performance liquid chromatography-mass spectrometry (UHPLC-MS) fingerprints of 10 batches of *Eupolyphaga steleophaga* enzymolysis polypeptides; Table S1. Characteristic-ion information of *Eupolyphaga steleophaga* enzymolysis polypeptides; Tables S2–S7: Method validation of retention time and peak area of *Eupolyphaga steleophaga* enzymolysis polypeptides; Tables S8 and S9: Retention time and peak area and their RSDs of 10 batches *Eupolyphaga steleophaga* enzymolysis polypeptides.

## References

[1] Zhang Y., Zhan Y., Zhang D., Dai B., Ma W., Qi J., Liu R., He L. (2014). *Eupolyphaga sinensis walker* displays inhibition on hepatocellular carcinoma through regulating cell growth and metastasis signaling. Sci. Rep..

[2] van Huis A. (2022). Edible insects: Challenges and prospects. Entomol. Res..

[3] Mancini S., Riccioli F., Tzompa-Sosa D.A., Moruzzo R., Schouteten J.J., Liu A., Li J., Menozzi D., Sogari G. (2024). Exploring the intention to consume whole vs processed edible insects: Insights from traditional and non-traditional entomophagy countries. J. Agric. Food Res..

[4] Chinese Pharmacopoeia Commission (2020). Pharmacopoeia of the People’s Republic of China.

[5] Zhu H.J., Xu T., Yan Y.M., Tu Z.C., Cheng Y.X. (2021). Neolignans and Norlignans from Insect Medicine Polyphaga plancyi and Their Biological Activities. Nat. Prod. Bioprospect..

[6] Fu X., Shao B.H., Wei X., Wang H.H., Chen X., Zhao T.T., Wang C.M. (2022). *Tubiechong*: A review on ethnomedicinal uses, bioactive chemical constituents and pharmacological activities. J. Ethnopharmacol..

[7] Xie J., Zhang D., Liu C., Wang L. (2021). A periodic review of chemical and pharmacological profiles of *Tubiechong* as insect Chinese medicine. RSC Adv..

[8] Yang X.N., Ruan L.J., Jiang X., Song Z.J., Wei K.H., Qin S.S., Liang Y., Hou X.L., Wang X.J., Miao J.H. (2022). Overview of research and development of polypeptide drugs and traditional Chinese medicine-peptides. Zhongguo Zhong Yao Za Zhi.

[9] Dolton G., Bulek A., Wall A., Thomas H., Hopkins J.R., Rius C., Galloway S.A., Whalley T., Tan L.R., Morin T. (2024). HLA A*24:02-restricted T cell receptors cross-recognize bacterial and preproinsulin peptides in type 1 diabetes. J. Clin. Investig..

[10] Allemann A., Staubli S.M., Nebiker C.A. (2024). Trypsin and Trypsinogen Activation Peptide in the Prediction of Severity of Acute Pancreatitis. Life.

[11] Zhang N., Zhao Y., Shi Y., Chen R., Fu X., Zhao Y. (2019). Polypeptides extracted from *Eupolyphaga sinensis walker* via enzymic digestion alleviate UV radiation-induced skin photoaging. Biomed. Pharmacother..

[12] Peng H., Wang J., Chen J., Peng Y., Wang X., Chen Y., Kaplan D.L., Wang Q. (2023). Challenges and opportunities in delivering oral peptides and proteins. Expert Opin. Drug Deliv..

[13] Jia Z., Zhu X., Zhou Y., Wu J., Cao M., Hu C., Yu L., Xu R., Chen Z. (2024). Polypeptides from traditional Chinese medicine: Comprehensive review of perspective towards cancer management. Int. J. Biol. Macromol..

[14] Wang F.X., Wu N., Wei J.T., Liu J., Zhao J., Ji A.G., Lin X.K. (2013). A novel protein from *Eupolyphaga sinensis* inhibits adhesion, migration, and invasion of human lung cancer A549 cells. Biochem. Cell Biol..

[15] Tang X.M., Guo J.L., Chen L., Ho P.C.L. (2020). Application for proteomics analysis technology in studying animal-derived traditional Chinese medicine: A review. J. Pharm. Biomed. Anal..

[16] Tao H.J. (1986). The Annotated Catalogue of Famous Physicians.

[17] Li S.Z. (2012). The Compendium of Materia Medica.

[18] Su J. (2004). Tang Materia Medica.

[19] Zhang Y. (2011). Identification of Eupolyphaga steleophaga and their adulterants. Chin. J. Clin. Res..

[20] Albenzio M., Santillo A., Caroprese M., Della Malva A., Marino R. (2017). Bioactive Peptides in Animal Food Products. Foods.

[21] Michelfelder A.J. (2009). Soy: A complete source of protein. Am. Fam. Physician.

[22] Toldrá F., Mora L. (2021). Proteins and Bioactive Peptides in High Protein Content Foods. Foods.

[23] Marciniak A., Suwal S., Naderi N., Pouliot Y., Doyen A. (2018). Enhancing enzymatic hydrolysis of food proteins and production of bioactive peptides using high hydrostatic pressure technology. Trends Food Sci. Technol..

[24] Akbarian M., Khani A., Eghbalpour S., Uversky V.N. (2022). Bioactive Peptides: Synthesis, Sources, Applications, and Proposed Mechanisms of Action. Int. J. Mol. Sci..

[25] Lee A.C., Harris J.L., Khanna K.K., Hong J.H. (2019). A Comprehensive Review on Current Advances in Peptide Drug Development and Design. Int. J. Mol. Sci..

[26] Lau J.L., Dunn M.K. (2018). Therapeutic peptides: Historical perspectives, current development trends, and future directions. Bioorganic Med. Chem..

[27] Ma Z., Mondor M., Goycoolea V.F., Hernández-Álvarez A.J. (2023). Current state of insect proteins: Extraction technologies, bioactive peptides and allergenicity of edible insect proteins. Food Funct..

[28] Kasicka V. (2010). Analysis of amino acids and peptides by separation and mass spectrometric methods. J. Sep. Sci..

[29] Li J., Lv X., Li B., Liu L., Yu C., Cheng H., Zhou J., Zhu Y., Ma H. (2022). Identification of peptides of cinobufacini by gel filter chromatography and peptidomics. J. Sep. Sci..

[30] Chen Y., Ren Y., Wang L., Huang Z. (2021). Analysis of A1-type and A2-type β-casein in Maiwa Yak and Pien-niu milk by HPLC-high-resolution MS and tandem MS. J. Sep. Sci..

[31] Shen W., Matsui T. (2017). Current knowledge of intestinal absorption of bioactive peptides. Food Funct..

[32] Wang H., Dong P., Liu X., Zhang Z., Li H., Li Y., Zhang J., Dai L., Wang S. (2022). Active Peptide AR-9 From *Eupolyphaga sinensis* Reduces Blood Lipid and Hepatic Lipid Accumulation by Restoring Gut Flora and Its Metabolites in a High Fat Diet-Induced Hyperlipidemia Rat. Front. Pharmacol..

[33] Shan J., Dong P., Li H., Xu J., Li H., Yu Y., Dai L., Gao P., Wang S., Zhang J. (2020). Study on lipid-lowering mechanism of active peptide DP17 from *Eupolyphaga steleophaga* in hyperlipidemia rats. Zhongguo Zhong Yao Za Zhi.

[34] Dong P., Wang H., Li Y., Yu J., Liu X., Wang Y., Dai L., Wang S. (2024). Active peptides from *Eupolyphaga sinensis walker* attenuates experimental hyperlipidemia by regulating the gut microbiota and biomarkers in rats with dyslipidemia. Biomed. Pharmacother..

[35] Ge G.F., Yu C.H., Yu B., Shen Z.H., Zhang D.L., Wu Q.F. (2012). Antitumor effects and chemical compositions of *Eupolyphaga sinensis Walker* ethanol extract. J. Ethnopharmacol..

[36] Kim B.S., Jin S., Park J.Y., Kim S.Y. (2022). Scoping review of the medicinal effects of *Eupolyphaga sinensis Walker* and the underlying mechanisms. J. Ethnopharmacol..

[37] Song H., Sun H., Fang H., Yang L., Zhao Q., Sun Y., Yan G., Han Y., Wang X. (2024). Fingerprint profile analysis of *hirudo* polypeptide based on UHPLC–MS and its application. Sep. Sci. Plus.

