# Supplementary material for: Fingerprint Profile Analysis of Eupolyphaga steleophaga Polypeptide Based on UHPLC-MS and Its Application
Source: Pharmaceuticals (Basel). 2025 Jan 26;18(2):166. doi: 10.3390/ph18020166 (PMC11858523; doi:10.3390/ph18020166)
Supplement: Supplementary file 1 [file pharmaceuticals-18-00166-s001.zip › pharmaceuticals-3420127-supplementary.pdf]

Supplementary materials of UHPLC-MS fingerprint of Eupolyphaga steleophaga enzymolysis polypeptides

- **Figures S1-S3. Method validation of UHPLC-MS fingerprints of Eupolyphaga steleophaga enzymolysis polypeptides**
- **Figu r e S4. UHPLC-MS base peak ion chromatogram of Eupolyphaga steleophaga enzymolysis polypeptides**
- **Figure S5. Characteristic mass spectrometry (MS) of Eupolyphaga steleophaga enzymolysis polypeptides**
- **Figure S6. Ultrahigh-performance liquid chromatography-mass spectrometry (UHPLC-MS) fingerprints of 10 batches of Eupolyphaga steleophaga enzymolysis polypeptides.**
- **Table S1. Characteristic-ion information of Eupolyphaga steleophaga enzymolysis polypeptides.**
- **Tables S2-S7. Method validation of retention time and peak area of Eupolyphaga steleophaga enzymolysis polypeptides**
- **Tables S8 and S9. Retention time and peak area and their RSDs of 10 batches Eupolyphaga steleophaga enzymolysis polypeptides**

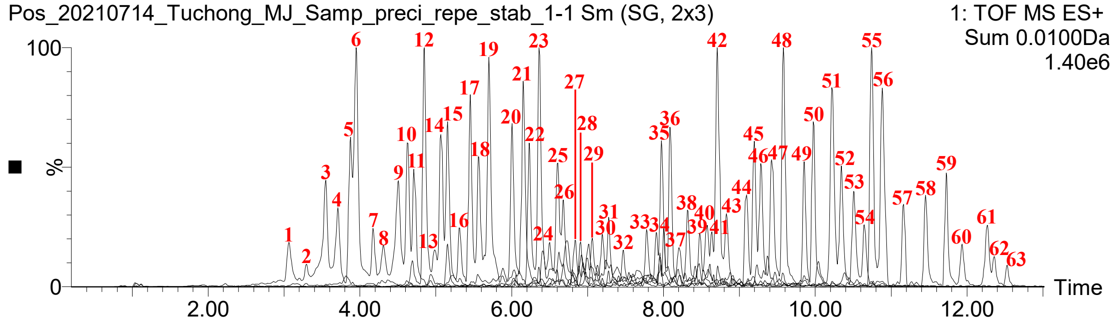

Figure S1. Precision investigation of UHPLC–MS fingerprint of Eupolyphaga steleophaga enzymolysis polypeptides

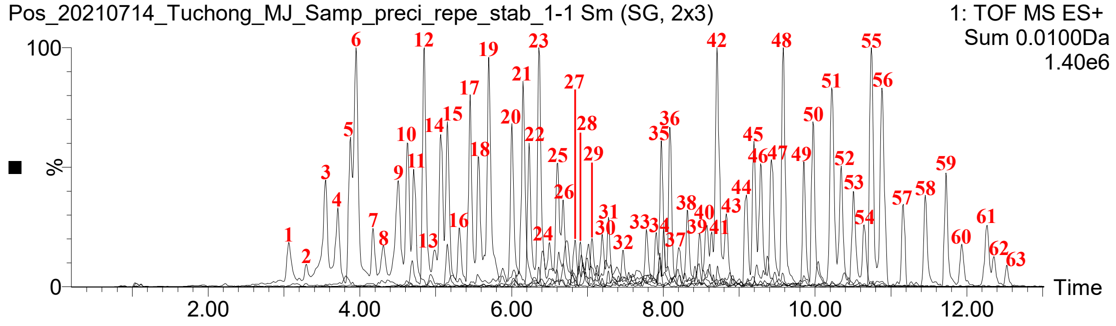

Figure S2. Repeatability investigation of UHPLC–MS fingerprint of Eupolyphaga steleophaga enzymolysis polypeptides

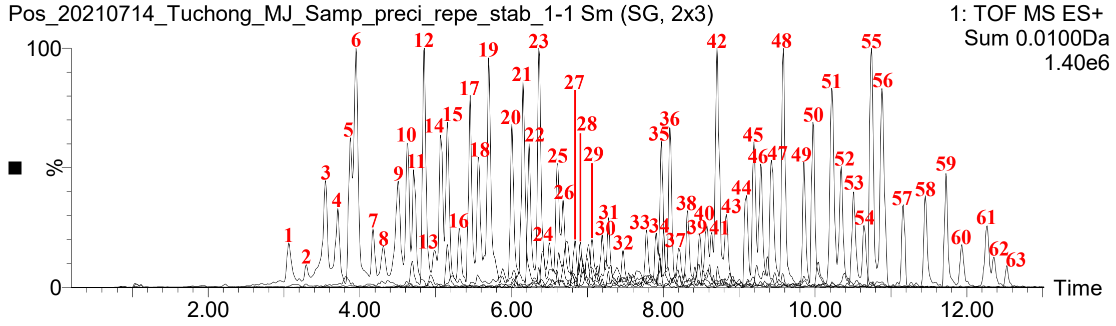

Figure S3. Stability investigation of UHPLC–MS fingerprint of Eupolyphaga steleophaga enzymolysis polypeptides

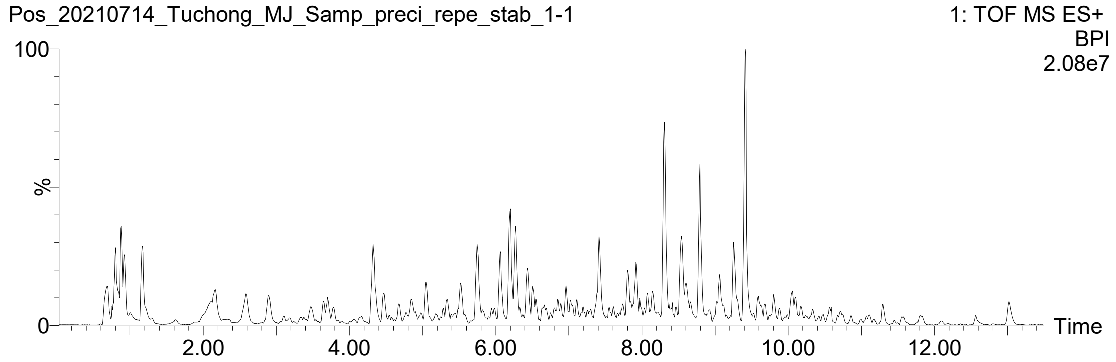

Figure S4. UHPLC–MS base peak ion chromatogram of of Eupolyphaga steleophaga enzymolysis polypeptides

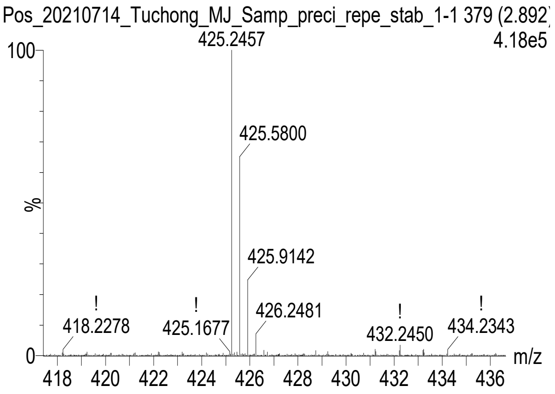
Pos_20210714_Tuchong_MJ_Samp_preci_repe_stab_1-1 379 (2.892)
4.18e5
425.2457
425.5800
425.9142
426.2481
418.2278
425.1677
432.2450
434.2343
100
%
0
m/z
418 420 422 424 426 428 430 432 434 436

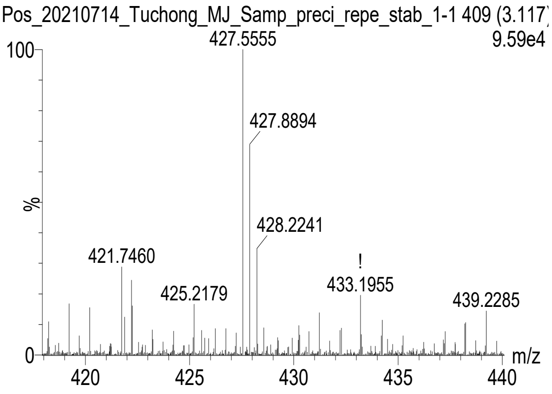
Pos_20210714_Tuchong_MJ_Samp_preci_repe_stab_1-1 409 (3.117)
9.59e4
427.5555
427.8894
428.2241
421.7460
425.2179
433.1955
439.2285
100
%
0
m/z
420 425 430 435 440

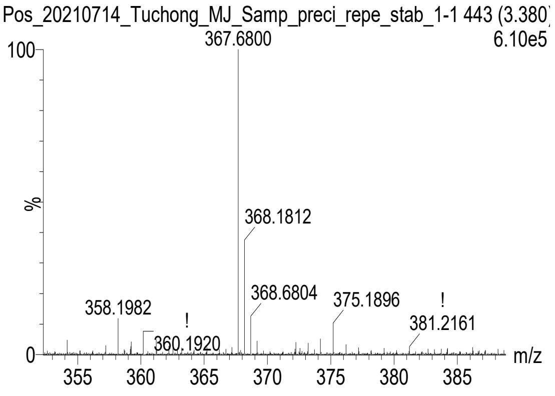
Pos_20210714_Tuchong_MJ_Samp_preci_repe_stab_1-1 443 (3.380)
6.10e5
367.6800
368.1812
368.6804
358.1982
360.1920
375.1896
381.2161
100
%
0
m/z
355 360 365 370 375 380 385

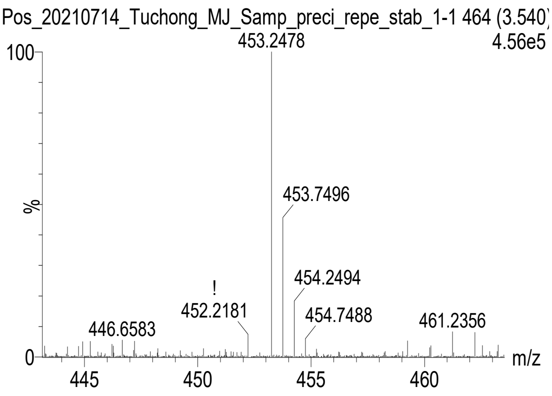
Pos_20210714_Tuchong_MJ_Samp_preci_repe_stab_1-1 464 (3.540)
4.56e5
453.2478
453.7496
454.2494
454.7488
446.6583
452.2181
461.2356
100
%
0
m/z
445 450 455 460

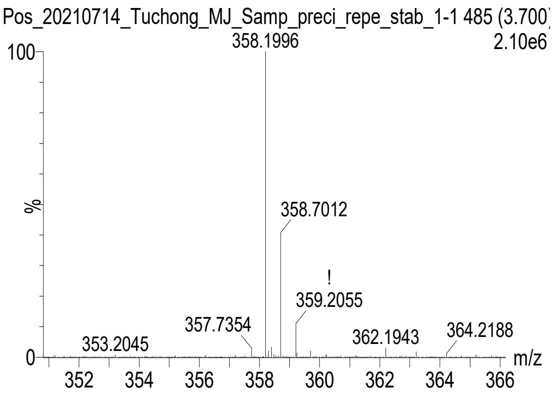
Pos_20210714_Tuchong_MJ_Samp_preci_repe_stab_1-1 485 (3.700)
2.10e6
358.1996
358.7012
359.2055
357.7354
353.2045
362.1943
364.2188
100
%
0
m/z
352 354 356 358 360 362 364 366

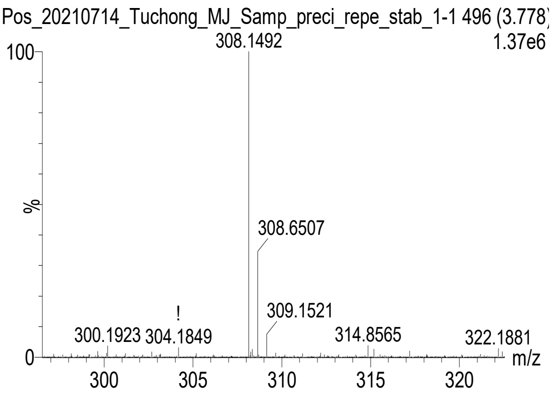
Pos_20210714_Tuchong_MJ_Samp_preci_repe_stab_1-1 496 (3.778)
1.37e6
308.1492
308.6507
309.1521
300.1923
304.1849
314.8565
322.1881
100
%
0
m/z
300 305 310 315 320

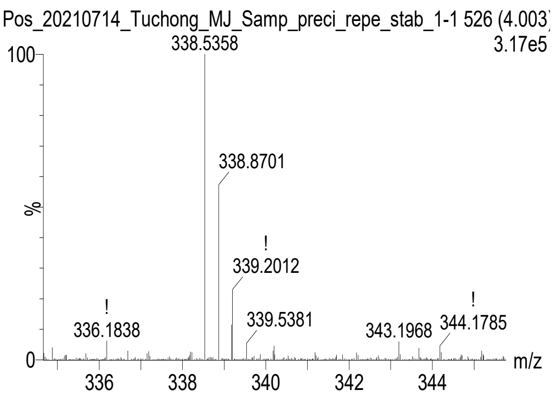
Pos_20210714_Tuchong_MJ_Samp_preci_repe_stab_1-1 526 (4.003)
3.17e5
338.5358
338.8701
339.2012
339.5381
336.1838
343.1968
344.1785
100
%
0
m/z
336 338 340 342 344

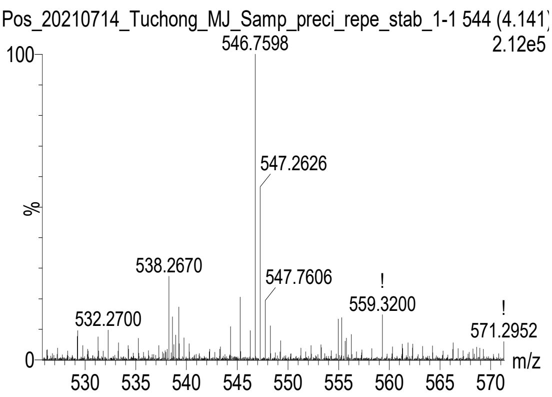
Pos_20210714_Tuchong_MJ_Samp_preci_repe_stab_1-1 544 (4.141)
2.12e5
546.7598
547.2626
547.7606
538.2670
532.2700
559.3200
571.2952
100
%
0
m/z
530 535 540 545 550 555 560 565 570

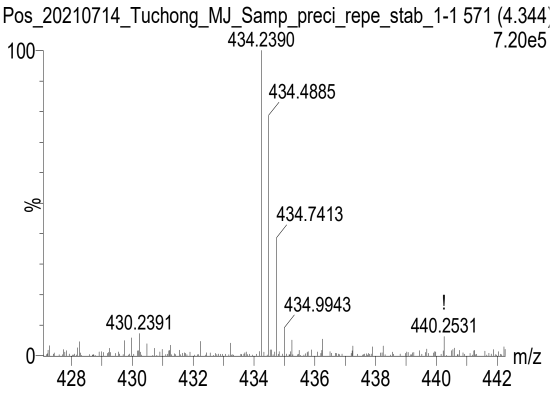
Pos_20210714_Tuchong_MJ_Samp_preci_repe_stab_1-1 571 (4.344)
434.2390
7.20e5
434.4885
434.7413
434.9943
430.2391
440.2531
100
%
0
m/z
428 430 432 434 436 438 440 442

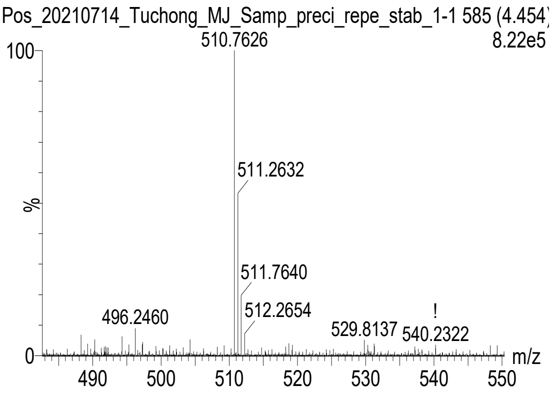
Pos_20210714_Tuchong_MJ_Samp_preci_repe_stab_1-1 585 (4.454)
510.7626
8.22e5
511.2632
511.7640
512.2654
496.2460
529.8137
540.2322
100
%
0
m/z
490 500 510 520 530 540 550

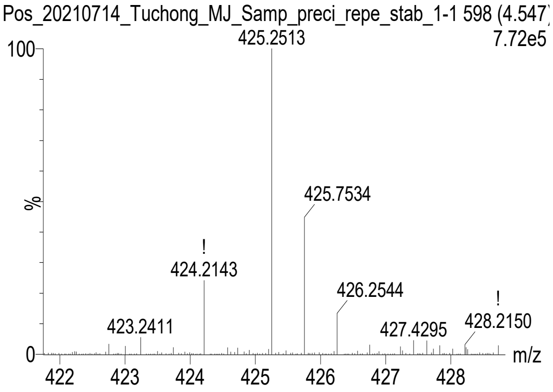
Pos_20210714_Tuchong_MJ_Samp_preci_repe_stab_1-1 598 (4.547)
425.2513
7.72e5
425.7534
424.2143
426.2544
423.2411
427.4295
428.2150
100
%
0
m/z
422 423 424 425 426 427 428

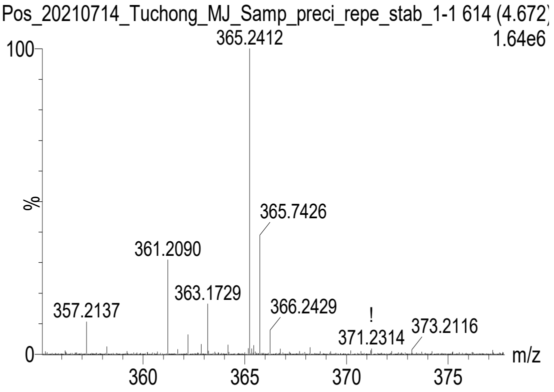
Pos_20210714_Tuchong_MJ_Samp_preci_repe_stab_1-1 614 (4.672)
365.2412
1.64e6
365.7426
361.2090
363.1729
357.2137
366.2429
371.2314
373.2116
100
%
0
m/z
360 365 370 375

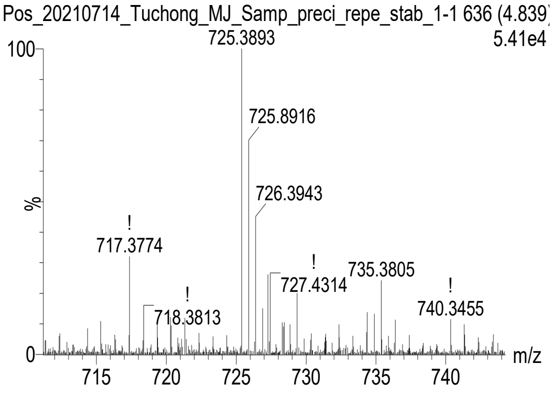
Pos_20210714_Tuchong_MJ_Samp_preci_repe_stab_1-1 636 (4.839)
725.3893
5.41e4
725.8916
726.3943
717.3774
727.4314
735.3805
740.3455
718.3813
100
%
0
m/z
715 720 725 730 735 740

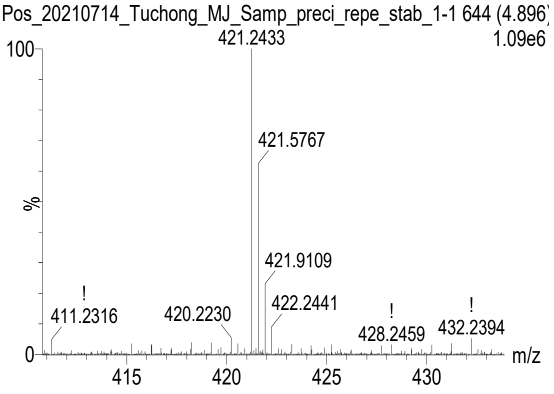
Pos_20210714_Tuchong_MJ_Samp_preci_repe_stab_1-1 644 (4.896)
421.2433
1.09e6
421.5767
421.9109
411.2316
420.2230
422.2441
428.2459
432.2394
100
%
0
m/z
415 420 425 430

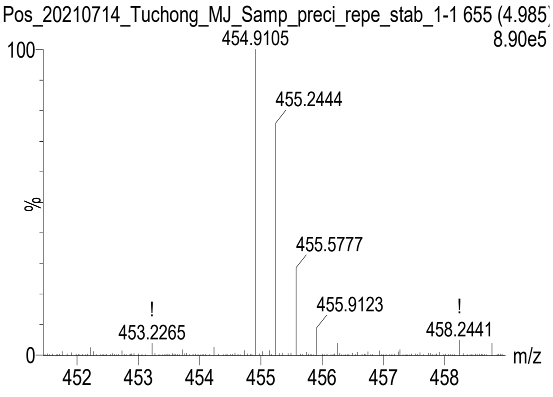
Pos_20210714_Tuchong_MJ_Samp_preci_repe_stab_1-1 655 (4.985)
454.9105
8.90e5
455.2444
455.5777
455.9123
453.2265
458.2441
100
%
0
m/z
452 453 454 455 456 457 458

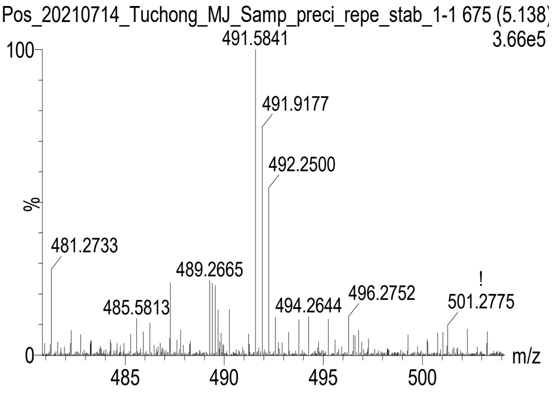
Pos_20210714_Tuchong_MJ_Samp_preci_repe_stab_1-1 675 (5.138)
491.5841
3.66e5
491.9177
492.2500
481.2733
489.2665
485.5813
494.2644
496.2752
501.2775
100
%
0
m/z
485 490 495 500

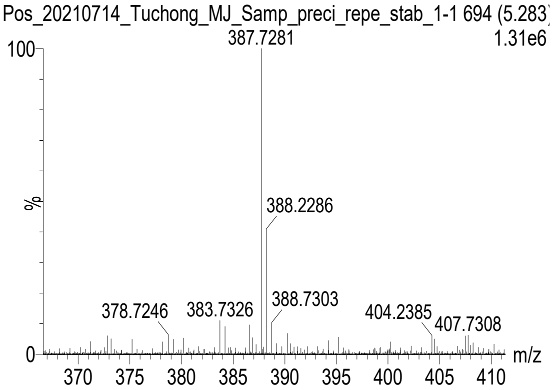
Pos_20210714_Tuchong_MJ_Samp_preci_repe_stab_1-1 694 (5.283)
1.31e6
387.7281
388.2286
378.7246
383.7326
388.7303
404.2385
407.7308
%
m/z

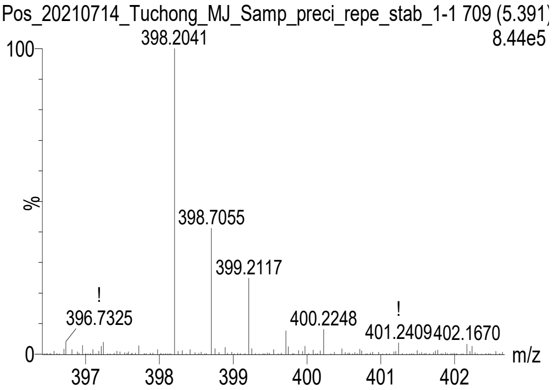
Pos_20210714_Tuchong_MJ_Samp_preci_repe_stab_1-1 709 (5.391)
8.44e5
398.2041
398.7055
399.2117
396.7325
400.2248
401.2409
402.1670
%
m/z

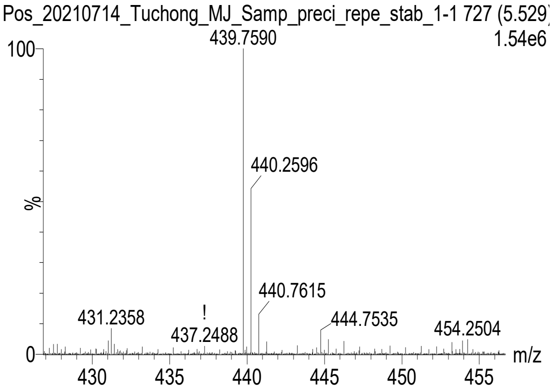
Pos_20210714_Tuchong_MJ_Samp_preci_repe_stab_1-1 727 (5.529)
1.54e6
439.7590
440.2596
440.7615
431.2358
437.2488
444.7535
454.2504
%
m/z

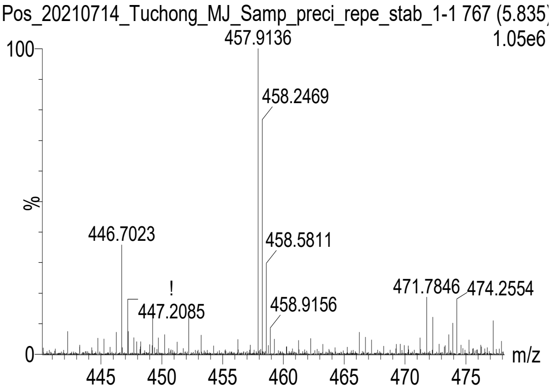
Pos_20210714_Tuchong_MJ_Samp_preci_repe_stab_1-1 767 (5.835)
1.05e6
457.9136
458.2469
446.7023
458.5811
447.2085
458.9156
471.7846
474.2554
%
m/z

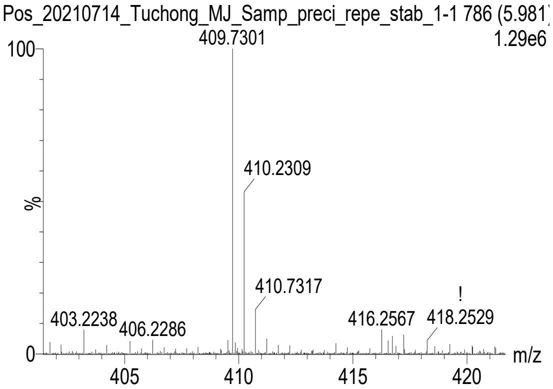
Pos_20210714_Tuchong_MJ_Samp_preci_repe_stab_1-1 786 (5.981)
1.29e6
409.7301
410.2309
410.7317
403.2238
406.2286
416.2567
418.2529
%
m/z

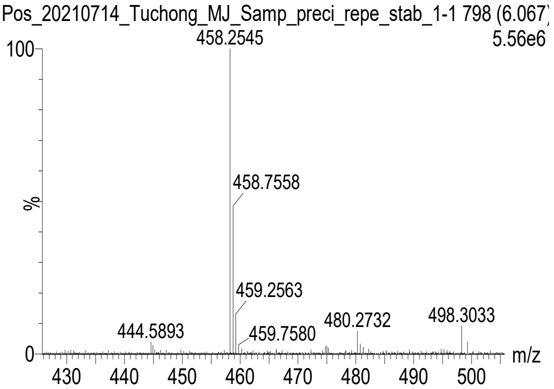
Pos_20210714_Tuchong_MJ_Samp_preci_repe_stab_1-1 798 (6.067)
5.56e6
458.2545
458.7558
459.2563
444.5893
459.7580
480.2732
498.3033
%
m/z

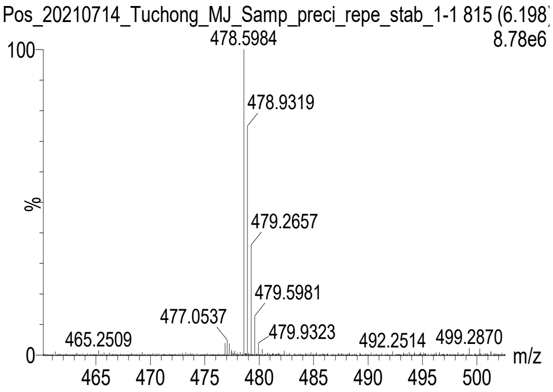
Pos_20210714_Tuchong_MJ_Samp_preci_repe_stab_1-1 815 (6.198)
8.78e6
478.5984
478.9319
479.2657
479.5981
477.0537
465.2509
479.9323
492.2514
499.2870
%
m/z

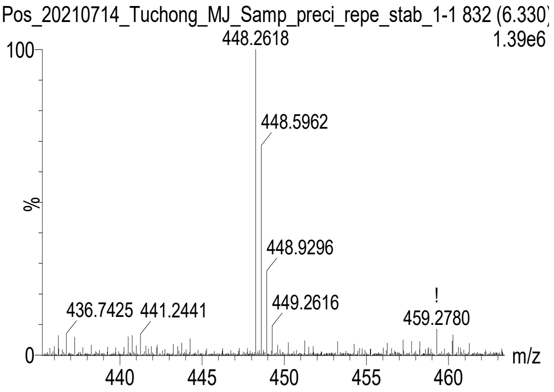
Pos_20210714_Tuchong_MJ_Samp_preci_repe_stab_1-1 832 (6.330)
1.39e6
448.2618
448.5962
448.9296
449.2616
436.7425
441.2441
459.2780
%
m/z

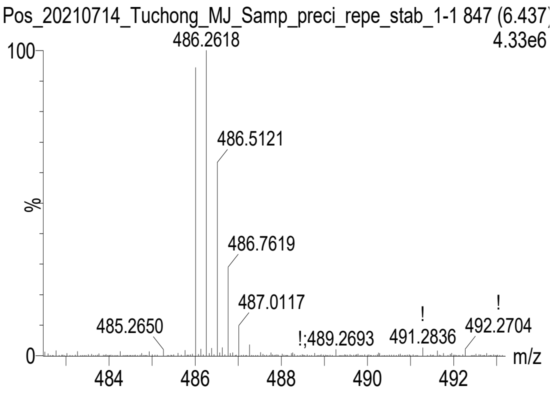
Pos_20210714_Tuchong_MJ_Samp_preci_repe_stab_1-1 847 (6.437)
4.33e6
486.2618
486.5121
486.7619
487.0117
485.2650
!;489.2693
491.2836
492.2704
%
m/z

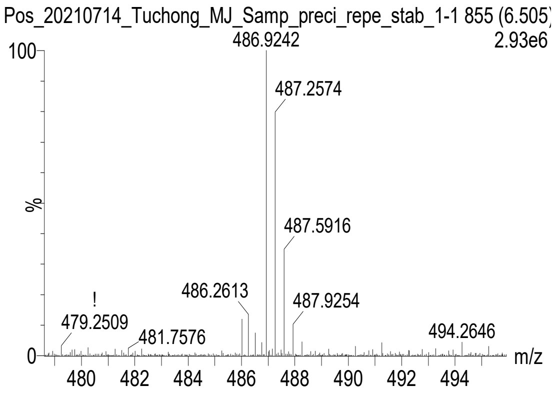
Pos_20210714_Tuchong_MJ_Samp_preci_repe_stab_1-1 855 (6.505)
2.93e6
486.9242
487.2574
487.5916
486.2613
487.9254
479.2509
481.7576
494.2646
%
m/z

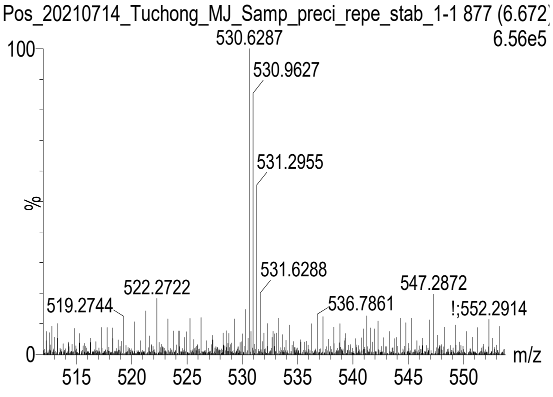
Pos_20210714_Tuchong_MJ_Samp_preci_repe_stab_1-1 877 (6.672)
6.56e5
530.6287
530.9627
531.2955
531.6288
519.2744
522.2722
536.7861
547.2872
!;552.2914
%
m/z

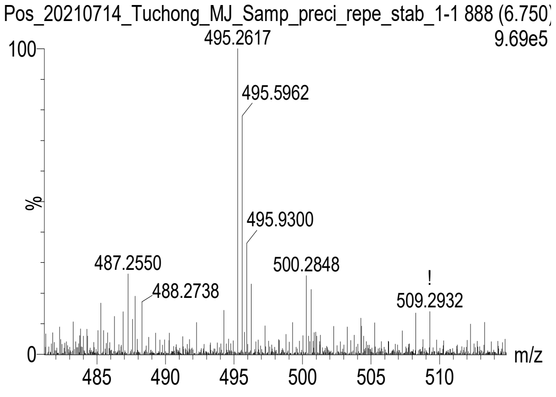
Pos_20210714_Tuchong_MJ_Samp_preci_repe_stab_1-1 888 (6.750)
9.69e5
495.2617
495.5962
495.9300
487.2550
488.2738
500.2848
509.2932
%
m/z

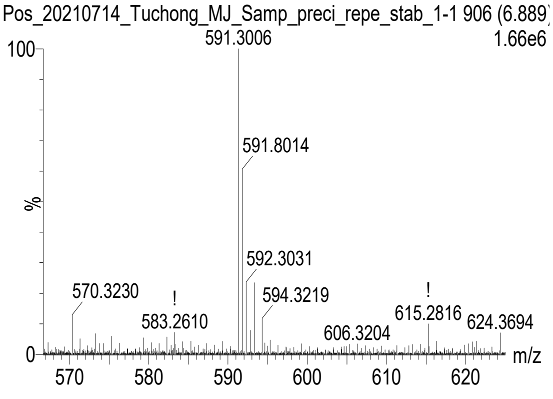
Pos_20210714_Tuchong_MJ_Samp_preci_repe_stab_1-1 906 (6.889)
1.66e6
591.3006
591.8014
592.3031
594.3219
570.3230
583.2610
606.3204
615.2816
624.3694
%
m/z

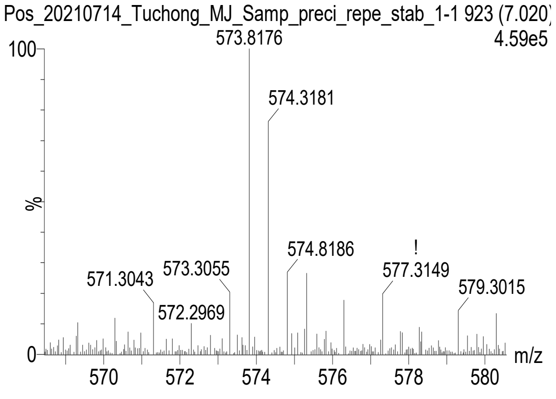
Pos_20210714_Tuchong_MJ_Samp_preci_repe_stab_1-1 923 (7.020)
4.59e5
573.8176
574.3181
574.8186
571.3043
573.3055
572.2969
577.3149
579.3015
%
m/z

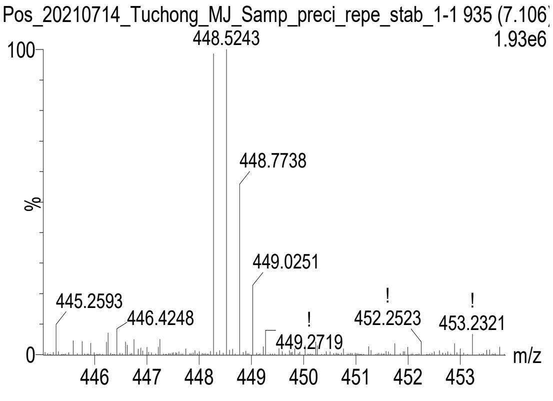
Pos_20210714_Tuchong_MJ_Samp_preci_repe_stab_1-1 935 (7.106)
1.93e6
448.5243
448.7738
449.0251
445.2593
446.4248
449.2719
452.2523
453.2321
%
m/z

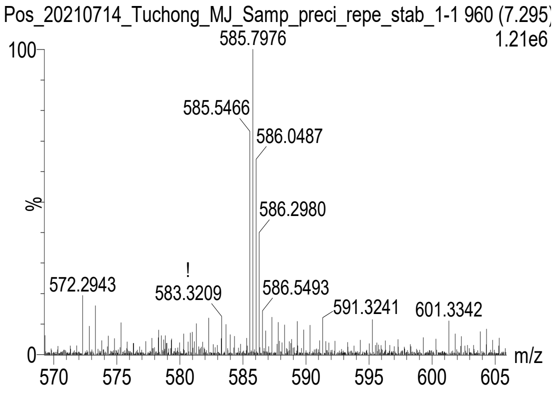
Pos_20210714_Tuchong_MJ_Samp_preci_repe_stab_1-1 960 (7.295)
1.21e6
585.7976
585.5466
586.0487
586.2980
572.2943
583.3209
586.5493
591.3241
601.3342
%
m/z

Pos_20210714_Tuchong_MJ_Samp_preci_repe_stab_1-1 1001 (7.60[illegible]

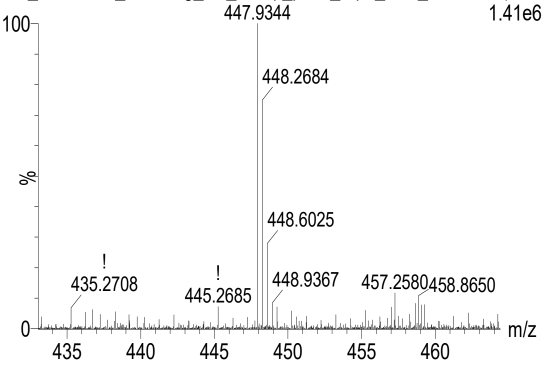

Pos_20210714_Tuchong_MJ_Samp_preci_repe_stab_1-1 1017 (7.73[illegible]

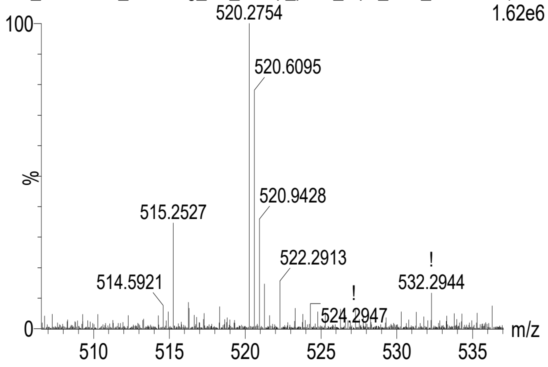

Pos_20210714_Tuchong_MJ_Samp_preci_repe_stab_1-1 1027 (7.80[illegible]

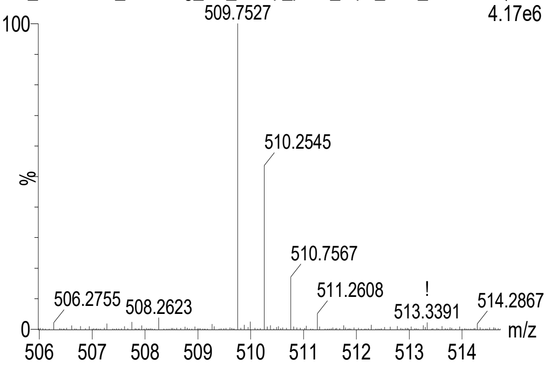

Pos_20210714_Tuchong_MJ_Samp_preci_repe_stab_1-1 1041 (7.91[illegible]

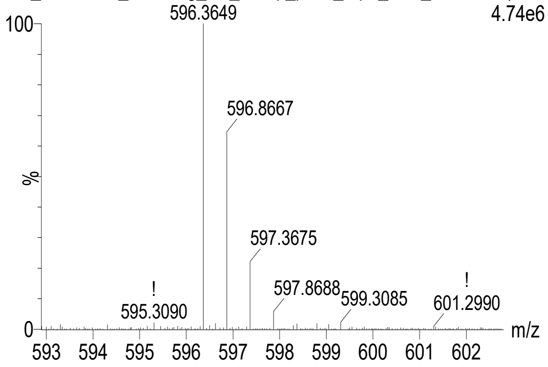

Pos_20210714_Tuchong_MJ_Samp_preci_repe_stab_1-1 1058 (8.03[illegible]

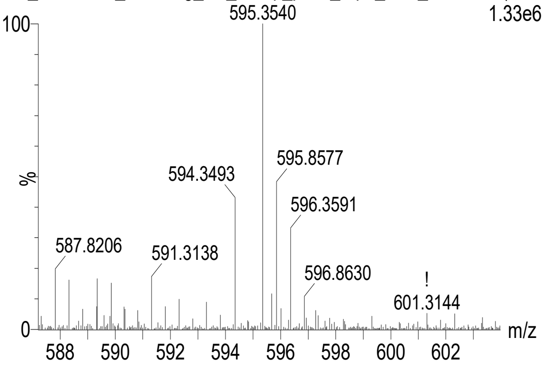

Pos_20210714_Tuchong_MJ_Samp_preci_repe_stab_1-1 1072 (8.14[illegible]

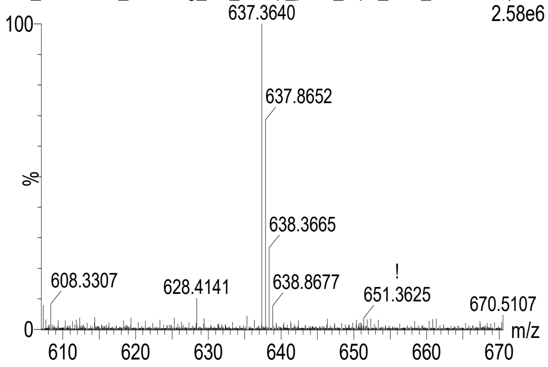

Pos_20210714_Tuchong_MJ_Samp_preci_repe_stab_1-1 1093 (8.30[illegible]

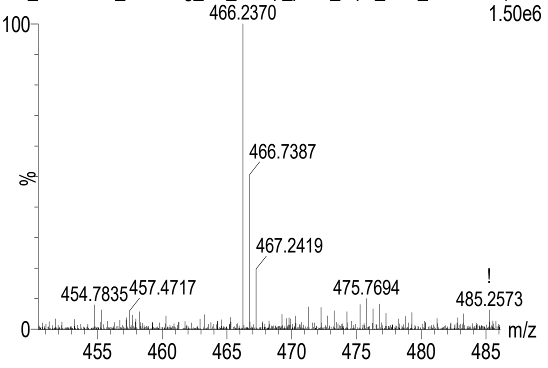

Pos_20210714_Tuchong_MJ_Samp_preci_repe_stab_1-1 1109 (8.43[illegible]

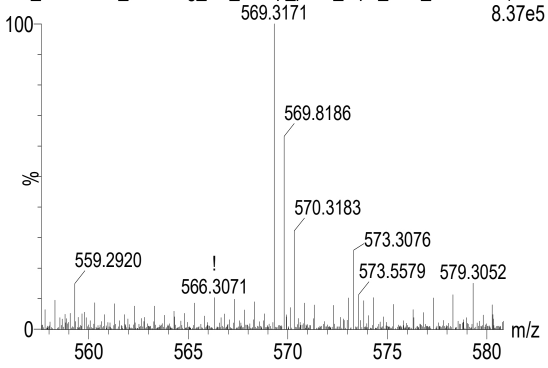

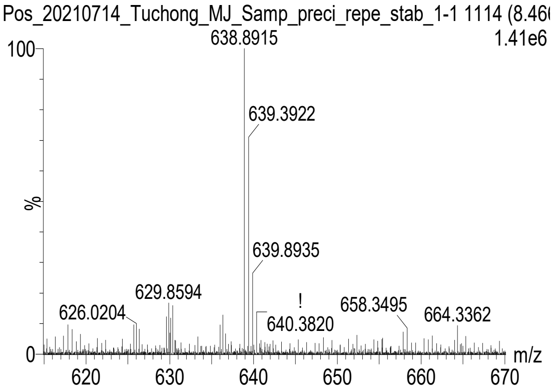
Pos_20210714_Tuchong_MJ_Samp_preci_repe_stab_1-1 1114 (8.46
638.8915
1.41e6
639.3922
639.8935
629.8594
626.0204
640.3820
658.3495
664.3362
m/z
%

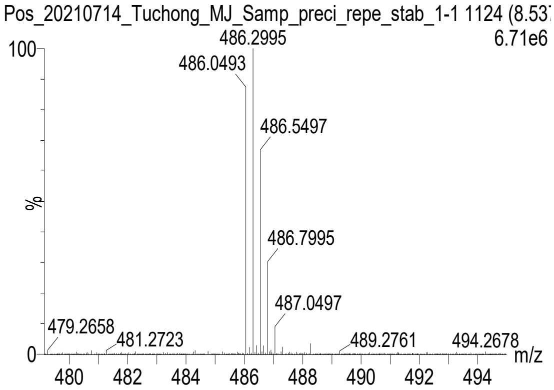
Pos_20210714_Tuchong_MJ_Samp_preci_repe_stab_1-1 1124 (8.53
486.2995
6.71e6
486.0493
486.5497
486.7995
487.0497
479.2658
481.2723
489.2761
494.2678
m/z
%

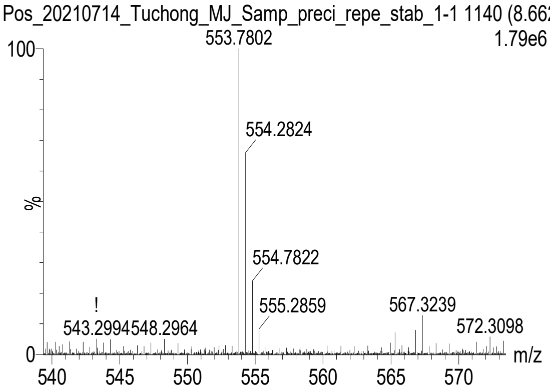
Pos_20210714_Tuchong_MJ_Samp_preci_repe_stab_1-1 1140 (8.66
553.7802
1.79e6
554.2824
554.7822
555.2859
567.3239
543.2994
548.2964
572.3098
m/z
%

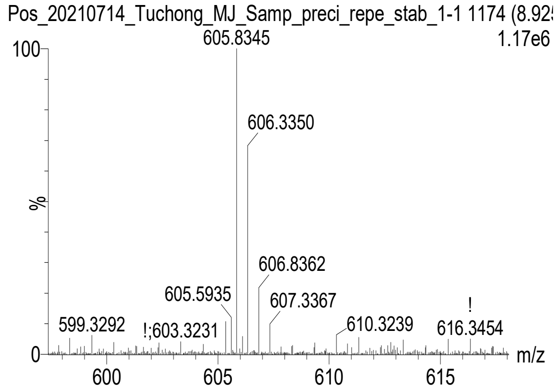
Pos_20210714_Tuchong_MJ_Samp_preci_repe_stab_1-1 1174 (8.92
605.8345
1.17e6
606.3350
606.8362
605.5935
607.3367
599.3292
603.3231
610.3239
616.3454
m/z
%

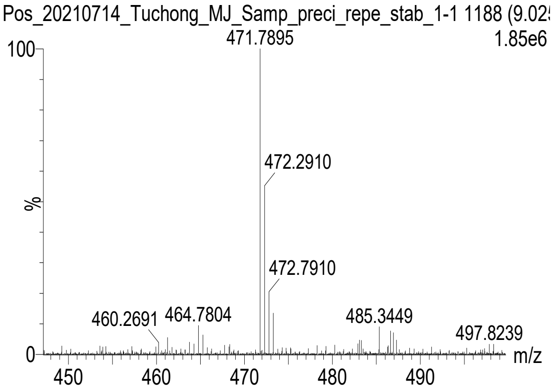
Pos_20210714_Tuchong_MJ_Samp_preci_repe_stab_1-1 1188 (9.02
471.7895
1.85e6
472.2910
472.7910
460.2691
464.7804
485.3449
497.8239
m/z
%

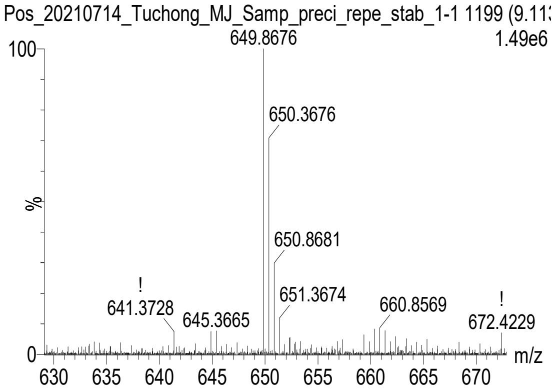
Pos_20210714_Tuchong_MJ_Samp_preci_repe_stab_1-1 1199 (9.11
649.8676
1.49e6
650.3676
650.8681
641.3728
645.3665
651.3674
660.8569
672.4229
m/z
%

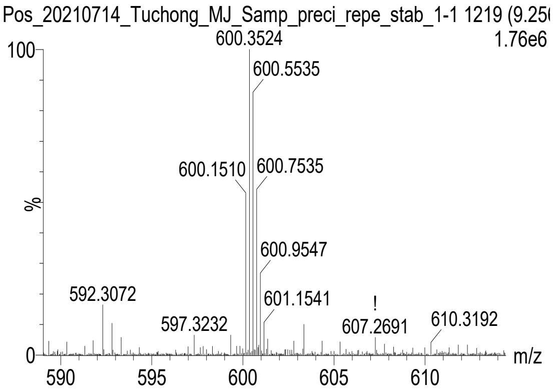
Pos_20210714_Tuchong_MJ_Samp_preci_repe_stab_1-1 1219 (9.25
600.3524
1.76e6
600.5535
600.1510
600.7535
600.9547
592.3072
601.1541
597.3232
607.2691
610.3192
m/z
%

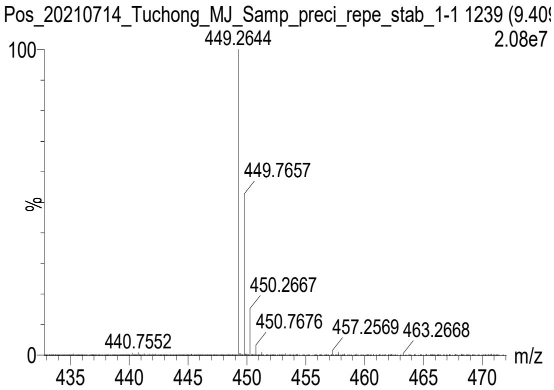
Pos_20210714_Tuchong_MJ_Samp_preci_repe_stab_1-1 1239 (9.40
449.2644
2.08e7
449.7657
450.2667
450.7676
457.2569
463.2668
440.7552
m/z
%

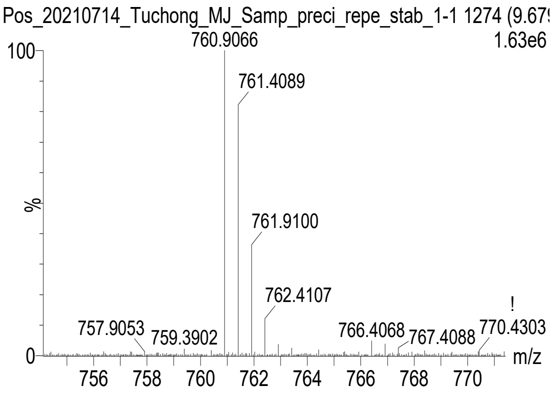
Pos_20210714_Tuchong_MJ_Samp_preci_repe_stab_1-1 1274 (9.67
760.9066
1.63e6
761.4089
761.9100
762.4107
757.9053
759.3902
766.4068
767.4088
770.4303
m/z
%

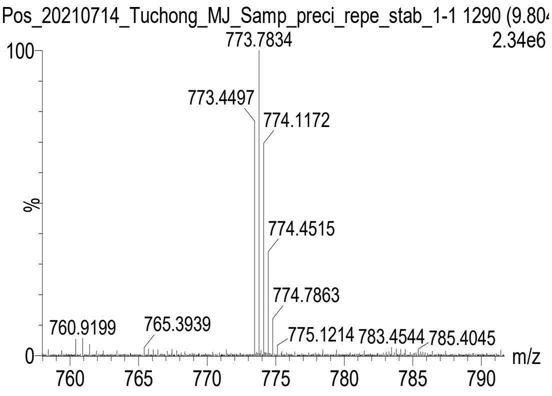
Pos_20210714_Tuchong_MJ_Samp_preci_repe_stab_1-1 1290 (9.80
773.7834
2.34e6
773.4497
774.1172
774.4515
774.7863
760.9199
765.3939
775.1214
783.4544
785.4045
m/z
%

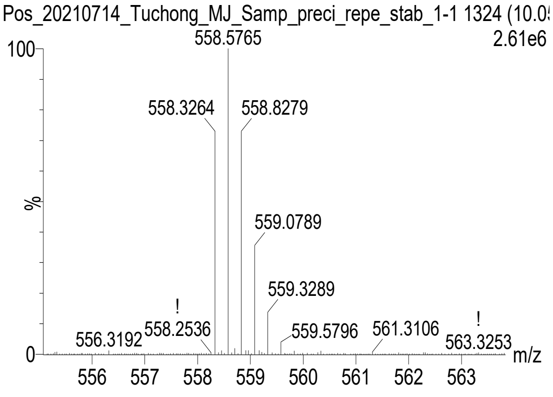
Pos_20210714_Tuchong_MJ_Samp_preci_repe_stab_1-1 1324 (10.0
558.5765
2.61e6
558.3264
558.8279
559.0789
559.3289
556.3192
558.2536
559.5796
561.3106
563.3253
m/z
%

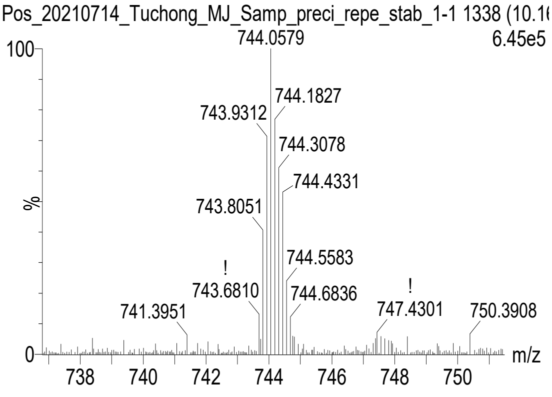
Pos_20210714_Tuchong_MJ_Samp_preci_repe_stab_1-1 1338 (10.1
744.0579
6.45e5
743.9312
744.1827
744.3078
744.4331
743.8051
744.5583
743.6810
744.6836
741.3951
747.4301
750.3908
m/z
%

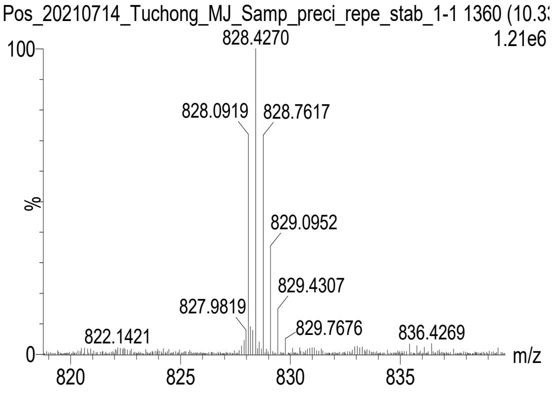
Pos_20210714_Tuchong_MJ_Samp_preci_repe_stab_1-1 1360 (10.3
828.4270
1.21e6
828.0919
828.7617
829.0952
829.4307
827.9819
822.1421
829.7676
836.4269
m/z
%

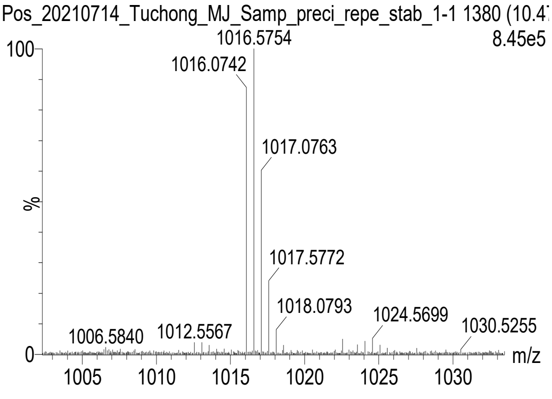
Pos_20210714_Tuchong_MJ_Samp_preci_repe_stab_1-1 1380 (10.4
1016.5754
8.45e5
1016.0742
1017.0763
1017.5772
1018.0793
1006.5840
1012.5567
1024.5699
1030.5255
m/z
%

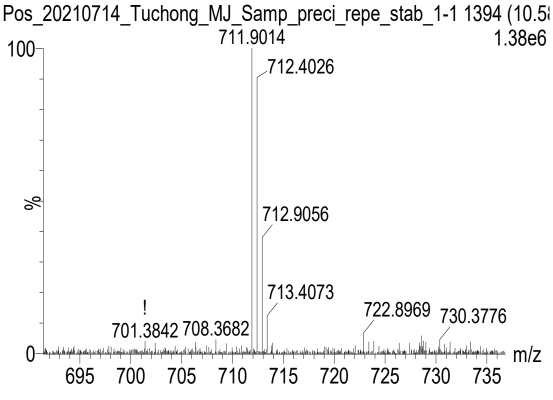
Pos_20210714_Tuchong_MJ_Samp_preci_repe_stab_1-1 1394 (10.5
711.9014
1.38e6
712.4026
712.9056
713.4073
701.3842
708.3682
722.8969
730.3776
m/z
%

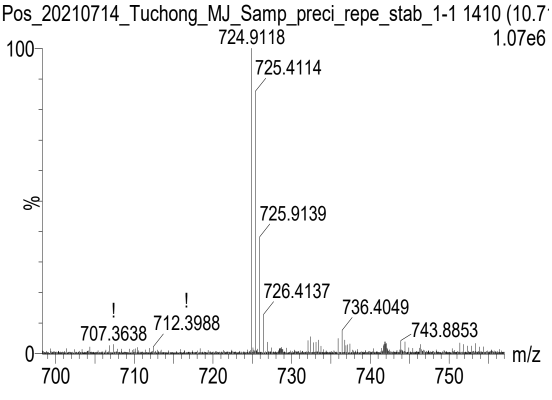
Pos_20210714_Tuchong_MJ_Samp_preci_repe_stab_1-1 1410 (10.7
724.9118
1.07e6
725.4114
725.9139
726.4137
707.3638
712.3988
736.4049
743.8853
m/z
%

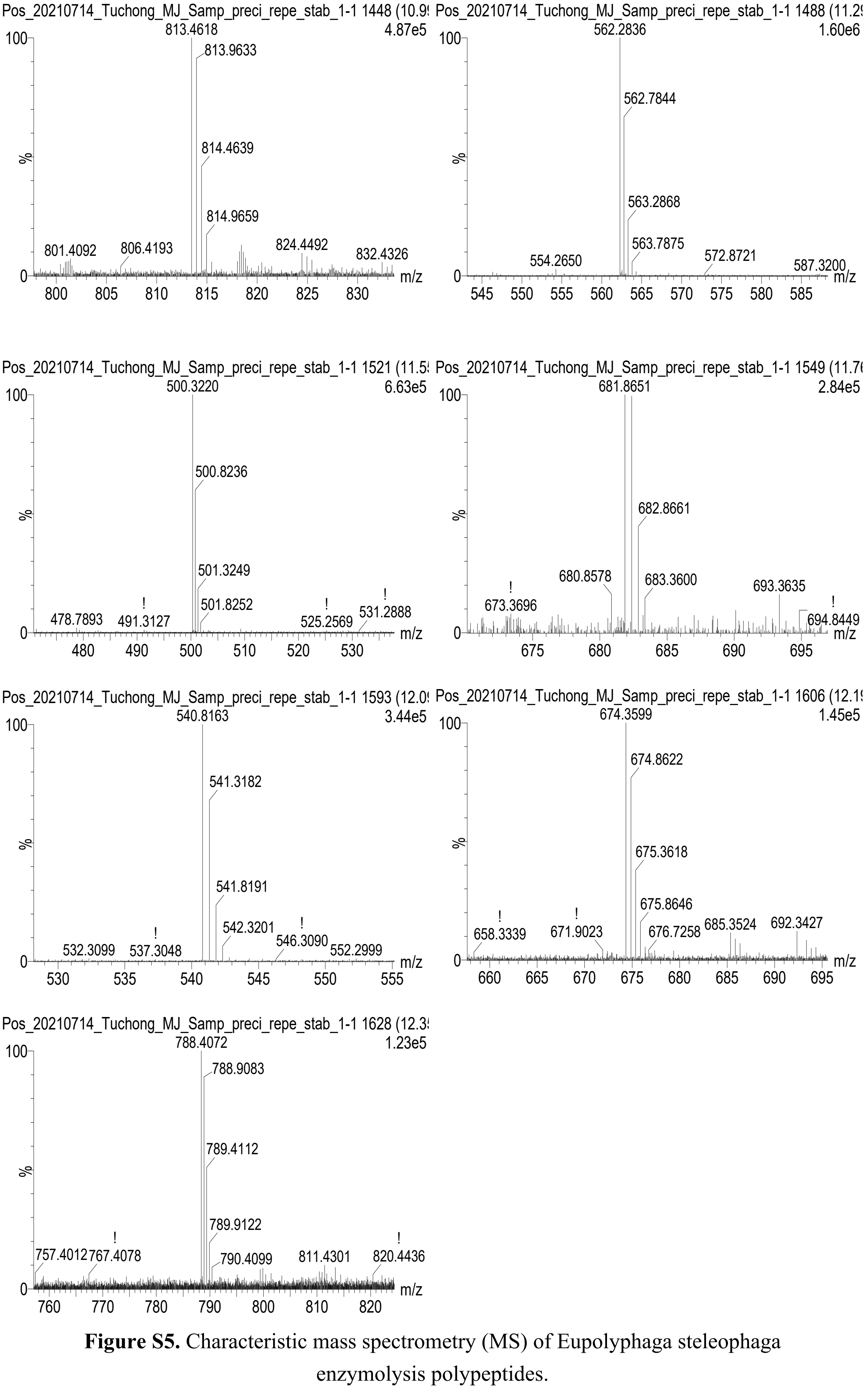

Figure S5. Characteristic mass spectrometry (MS) of Eupolyphaga steleophaga enzymolysis polypeptides.

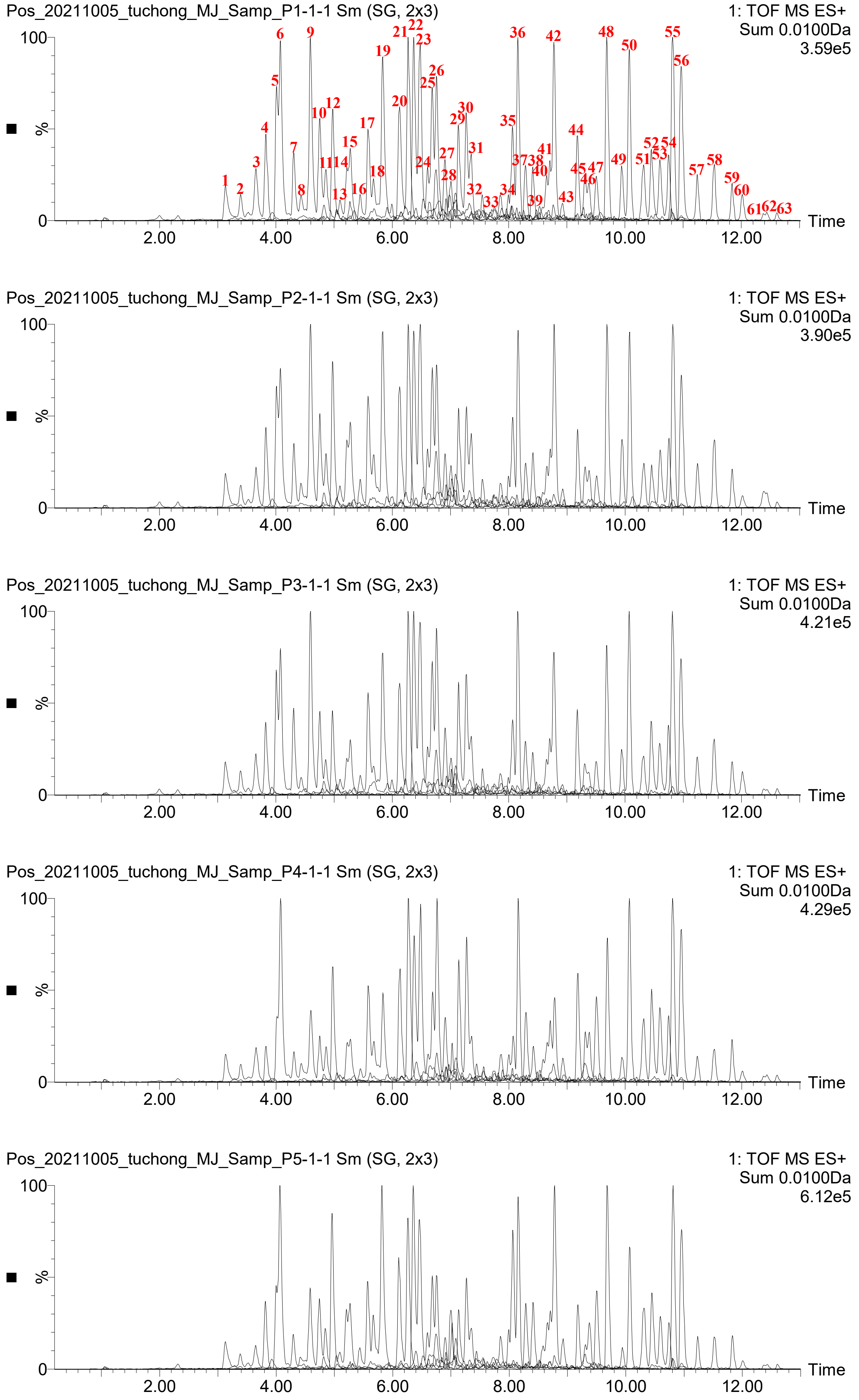
Pos_20211005_tuchong_MJ_Samp_P1-1-1 Sm (SG, 2x3)
1: TOF MS ES+
Sum 0.0100Da
3.59e5
1 2 3 4 5 6 7 8 9 10 11 12 13 14 15 16 17 18 19 20 21 22 23 24 25 26 27 28 29 30 31 32 33 34 35 36 37 38 39 40 41 42 43 44 45 46 47 48 49 50 51 52 53 54 55 56 57 58 59 60 61 62 63
Pos_20211005_tuchong_MJ_Samp_P2-1-1 Sm (SG, 2x3)
1: TOF MS ES+
Sum 0.0100Da
3.90e5
Pos_20211005_tuchong_MJ_Samp_P3-1-1 Sm (SG, 2x3)
1: TOF MS ES+
Sum 0.0100Da
4.21e5
Pos_20211005_tuchong_MJ_Samp_P4-1-1 Sm (SG, 2x3)
1: TOF MS ES+
Sum 0.0100Da
4.29e5
Pos_20211005_tuchong_MJ_Samp_P5-1-1 Sm (SG, 2x3)
1: TOF MS ES+
Sum 0.0100Da
6.12e5
100
%
0
Time
2.00
4.00
6.00
8.00
10.00
12.00

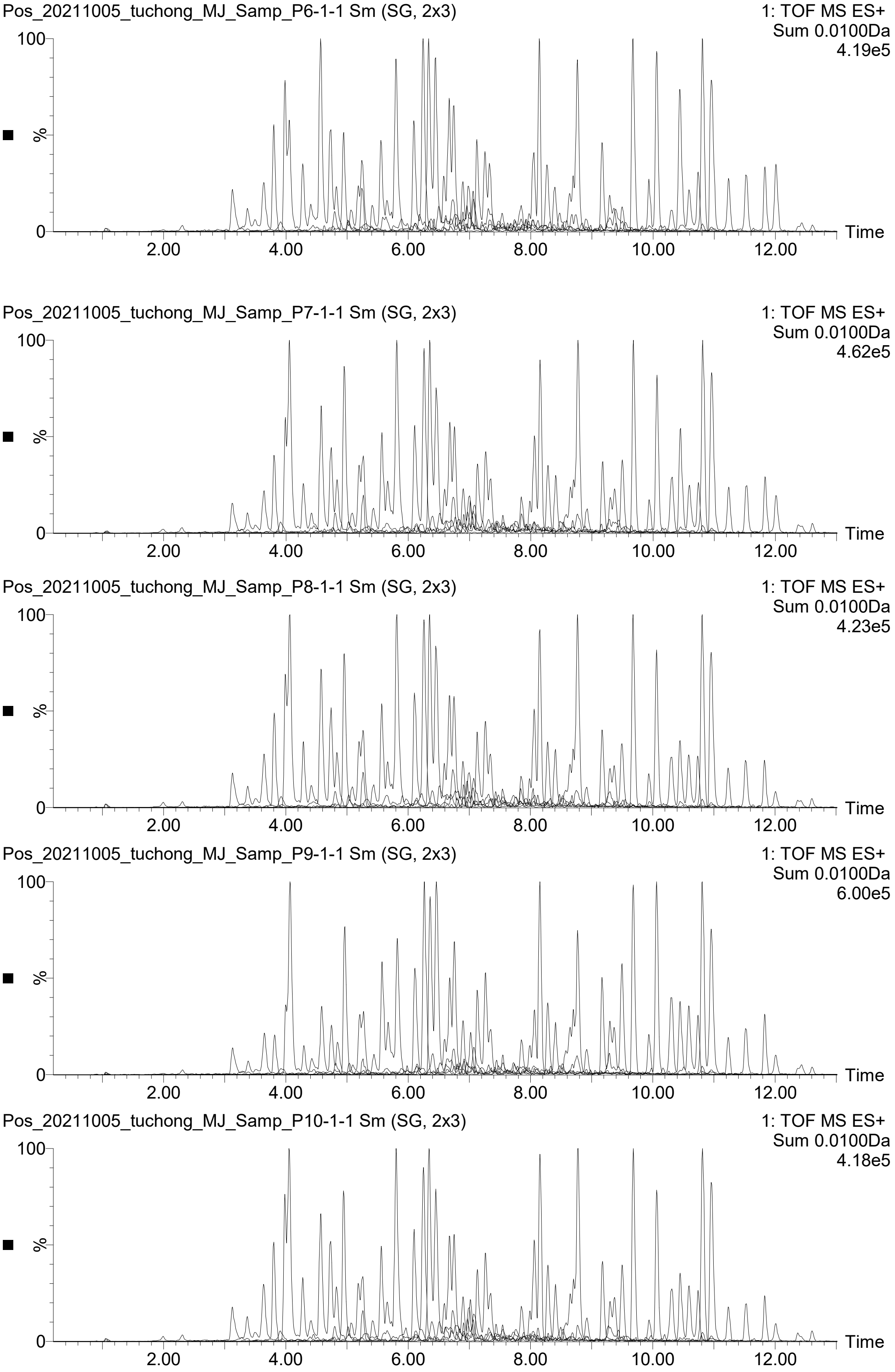

Figure S6. Ultrahigh-performance liquid chromatography-mass spectrometry (UHPLC-MS) fingerprints of 10 batches of Eupolyphaga steleophaga enzymolysis polypeptides.

Table S1. Characteristic-ion information of Eupolyphaga steleophaga enzymolysis polypeptides

Peak No.	Rt(min)	m/z	Ionic mode	Molecular Weight(Da)
1	2.89	425.2457	$[M + 3H]^{3+}$	1272.7371
2	3.12	427.5549	$[M + 3H]^{3+}$	1279.6647
3	3.38	367.6800	$[M + 2H]^{2+}$	733.3600
4	3.54	453.2478	$[M + 2H]^{2+}$	904.4956
5	3.70	358.1996	$[M + 2H]^{2+}$	714.3992
6	3.78	308.1492	$[M + 2H]^{2+}$	614.2984
7	4.00	338.5358	$[M + 3H]^{3+}$	1012.6074
8	4.14	546.7598	$[M + 2H]^{2+}$	1091.5196
9	4.34	434.2390	$[M + 4H]^{4+}$	1732.9560
10	4.45	510.7626	$[M + 2H]^{2+}$	1019.5252
11	4.55	425.2513	$[M + 2H]^{2+}$	848.5026
12	4.67	365.2412	$[M + 2H]^{2+}$	728.4824
13	4.84	725.3893	$[M + 2H]^{2+}$	1448.7786
14	4.90	421.2433	$[M + 3H]^{3+}$	1260.7299
15	4.98	454.9105	$[M + 3H]^{3+}$	1361.7315
16	5.14	491.5841	$[M + 3H]^{3+}$	1471.7523
17	5.28	387.7281	$[M + 2H]^{2+}$	773.4562
18	5.39	398.2041	$[M + 2H]^{2+}$	794.4082
19	5.53	439.7590	$[M + 2H]^{2+}$	877.5180
20	5.83	457.9136	$[M + 3H]^{3+}$	1370.7408
21	5.98	409.7301	$[M + 2H]^{2+}$	817.4602
22	6.07	458.2545	$[M + 2H]^{2+}$	914.5090
23	6.20	478.5984	$[M + 3H]^{3+}$	1432.7952
24	6.33	448.2618	$[M + 3H]^{3+}$	1341.7854
25	6.44	486.2618	$[M + 4H]^{4+}$	1941.0472
26	6.50	486.9242	$[M + 3H]^{3+}$	1457.7726
27	6.67	530.6287	$[M + 3H]^{3+}$	1588.8861

28	6.75	495.2617	$[M + 3H]^{3+}$	1482.7851
29	6.89	591.3006	$[M + 2H]^{2+}$	1180.6012
30	7.01	573.8171	$[M + 2H]^{2+}$	1145.6342
31	7.11	448.5243	$[M + 4H]^{4+}$	1790.0972
32	7.29	585.7976	$[M + 4H]^{4+}$	2339.1904
33	7.61	447.9344	$[M + 3H]^{3+}$	1340.8032
34	7.73	520.2754	$[M + 3H]^{3+}$	1557.8262
35	7.80	509.7527	$[M + 2H]^{2+}$	1017.5054
36	7.91	596.3649	$[M + 2H]^{2+}$	1190.7298
37	8.04	595.3540	$[M + 2H]^{2+}$	1188.7080
38	8.15	637.3640	$[M + 2H]^{2+}$	1272.7280
39	8.31	466.2370	$[M + 2H]^{2+}$	930.4740
40	8.43	569.3171	$[M + 2H]^{2+}$	1136.6342
41	8.47	638.8915	$[M + 2H]^{2+}$	1275.7830
42	8.54	486.2995	$[M + 4H]^{4+}$	1941.1980
43	8.66	553.7802	$[M + 2H]^{2+}$	1105.5604
44	8.92	605.8345	$[M + 2H]^{2+}$	1209.6690
45	9.02	471.7895	$[M + 2H]^{2+}$	941.5790
46	9.11	649.8676	$[M + 2H]^{2+}$	1297.7352
47	9.26	600.3524	$[M + 5H]^{5+}$	2996.7620
48	9.41	449.2644	$[M + 2H]^{2+}$	896.5288
49	9.68	760.9066	$[M + 2H]^{2+}$	1519.8132
50	9.80	773.7834	$[M + 3H]^{3+}$	2318.3502
51	10.06	558.5765	$[M + 4H]^{4+}$	2230.3060
52	10.17	744.0579	$[M + 8H]^{8+}$	5944.4632
53	10.33	828.4270	$[M + 3H]^{3+}$	2482.2810
54	10.48	1016.5754	$[M + 2H]^{2+}$	2031.1508
55	10.59	711.9014	$[M + 2H]^{2+}$	1421.8028
56	10.71	724.9118	$[M + 2H]^{2+}$	1447.8236

57	10.99	813.4618	$[M + 2H]^{2+}$	1624.9236
58	11.30	562.2836	$[M + 2H]^{2+}$	1122.5672
59	11.55	500.3220	$[M + 2H]^{2+}$	998.6440
60	11.76	681.8651	$[M + 2H]^{2+}$	1361.7302
61	12.10	540.8163	$[M + 2H]^{2+}$	1079.6326
62	12.18	674.3601	$[M + 2H]^{2+}$	1346.7202
63	12.36	788.4072	$[M + 2H]^{2+}$	1574.8144

Table S2. Precision investigation of Eupolyphaga steleophaga enzymolysis polypeptides retention time

**Peak No.**	**Continuous injection times**						**RSD%**
	1	2	3	4	5	6	
**1**	2.89	2.89	2.90	2.90	2.89	2.89	**0.12**
**2**	3.12	3.12	3.12	3.11	3.12	3.12	**0.18**
**3**	3.38	3.38	3.38	3.37	3.38	3.38	**0.09**
**4**	3.54	3.54	3.54	3.53	3.53	3.53	**0.20**
**5**	3.70	3.70	3.71	3.70	3.70	3.70	**0.08**
**6**	3.78	3.78	3.78	3.78	3.78	3.78	**0.02**
**7**	4.00	4.00	4.00	3.99	4.00	4.00	**0.16**
**8**	4.14	4.14	4.13	4.13	4.13	4.14	**0.08**
**9**	4.34	4.34	4.34	4.31	4.32	4.34	**0.33**
**10**	4.45	4.45	4.45	4.45	4.45	4.46	**0.01**
**11**	4.54	4.54	4.54	4.54	4.54	4.54	**0.01**
**12**	4.67	4.67	4.67	4.66	4.67	4.67	**0.07**
**13**	4.85	4.83	4.84	4.84	4.84	4.84	**0.10**
**14**	4.90	4.90	4.90	4.89	4.89	4.89	**0.08**
**15**	4.99	4.98	4.99	4.98	4.98	4.98	**0.07**
**16**	5.14	5.14	5.14	5.14	5.14	5.14	**0.01**
**17**	5.28	5.28	5.28	5.28	5.28	5.28	**0.06**
**18**	5.39	5.39	5.39	5.39	5.39	5.39	**0.01**

**19**	5.53	5.53	5.53	5.52	5.52	5.52	**0.10**
**20**	5.84	5.83	5.83	5.82	5.83	5.83	**0.09**
**21**	5.98	5.98	5.98	5.97	5.97	5.98	**0.06**
**22**	6.07	6.06	6.06	6.05	6.05	6.05	**0.09**
**23**	6.20	6.18	6.19	6.18	6.19	6.19	**0.09**
**24**	6.33	6.32	6.32	6.32	6.32	6.32	**0.05**
**25**	6.44	6.44	6.43	6.43	6.43	6.43	**0.05**
**26**	6.51	6.50	6.50	6.51	6.50	6.51	**0.02**
**27**	6.67	6.67	6.66	6.67	6.66	6.67	**0.06**
**28**	6.75	6.75	6.74	6.75	6.74	6.75	**0.06**
**29**	6.88	6.89	6.89	6.88	6.88	6.88	**0.05**
**30**	7.02	7.03	7.02	7.02	7.02	7.02	**0.03**
**31**	7.11	7.11	7.11	7.11	7.11	7.11	**0.05**
**32**	7.30	7.29	7.29	7.29	7.29	7.30	**0.04**
**33**	7.61	7.60	7.61	7.60	7.61	7.60	**0.05**
**34**	7.73	7.73	7.73	7.73	7.73	7.74	**0.05**
**35**	7.80	7.80	7.80	7.80	7.80	7.80	**0.04**
**36**	7.91	7.91	7.91	7.91	7.91	7.92	**0.03**
**37**	8.04	8.03	8.04	8.03	8.03	8.04	**0.05**
**38**	8.15	8.14	8.15	8.15	8.14	8.15	**0.08**
**39**	8.31	8.31	8.31	8.31	8.31	8.31	**0.03**
**40**	8.42	8.38	8.38	8.39	8.38	8.39	**0.15**
**41**	8.47	8.46	8.46	8.46	8.46	8.45	**0.05**
**42**	8.54	8.53	8.53	8.53	8.53	8.53	**0.04**
**43**	8.65	8.66	8.66	8.66	8.66	8.66	**0.04**
**44**	8.93	8.91	8.91	8.91	8.91	8.91	**0.08**
**45**	9.03	9.02	9.02	9.03	9.02	9.02	**0.06**
**46**	9.11	9.11	9.11	9.11	9.11	9.12	**0.04**
**47**	9.26	9.25	9.26	9.25	9.25	9.26	**0.04**

**48**	9.40	9.41	9.41	9.41	9.41	9.40	**0.04**
**49**	9.68	9.68	9.68	9.68	9.68	9.68	**0.02**
**50**	9.80	9.80	9.80	9.81	9.80	9.81	**0.02**
**51**	10.06	10.05	10.04	10.05	10.05	10.05	**0.05**
**52**	10.17	10.17	10.17	10.17	10.17	10.17	**0.03**
**53**	10.33	10.33	10.33	10.34	10.33	10.34	**0.02**
**54**	10.48	10.48	10.47	10.47	10.47	10.47	**0.03**
**55**	10.59	10.56	10.57	10.56	10.57	10.57	**0.09**
**56**	10.71	10.71	10.71	10.71	10.71	10.73	**0.07**
**57**	10.99	10.99	10.99	10.99	10.99	10.99	**0.02**
**58**	11.28	11.28	11.29	11.28	11.28	11.27	**0.05**
**59**	11.55	11.55	11.55	11.55	11.55	11.55	**0.02**
**60**	11.76	11.75	11.75	11.75	11.75	11.75	**0.05**
**61**	12.09	12.10	12.09	12.09	12.08	12.10	**0.04**
**62**	12.19	12.20	12.19	12.18	12.18	12.19	**0.08**
**63**	12.36	12.35	12.35	12.35	12.35	12.35	**0.03**

Table S3. Precision investigation of Eupolyphaga steleophaga enzymolysis polypeptides peak area

**Peak No.**	**Continuous injection times**						**RSD%**
	1	2	3	4	5	6	
**1**	15769	14811	15715	15109	13772	13150	**7.20**
**2**	4954	4235	4591	5093	4832	4916	**6.50**
**3**	48774	49558	49221	48892	41442	41884	**8.27**
**4**	29927	31385	29999	28285	26860	26532	**6.68**
**5**	39356	33651	31767	37450	33011	33952	**8.33**
**6**	87318	87870	87896	86823	84290	79976	**3.62**
**7**	12313	12737	12431	12673	12020	12041	**2.47**
**8**	14582	12204	13601	12579	11671	11377	**9.61**
**9**	41898	50782	47493	46724	48645	43107	**7.25**

**10**	42366	45589	41821	43680	43029	39612	**4.67**
**11**	34751	34531	32947	35369	35987	35461	**3.06**
**12**	82436	77325	79161	83112	79460	81436	**2.74**
**13**	9244	8914	9514	11320	10130	9862	**8.62**
**14**	55770	54725	62718	52784	52868	65709	**9.50**
**15**	53217	55666	53678	53004	49020	45909	**6.93**
**16**	15630	18237	16145	17125	15951	16305	**5.79**
**17**	59859	59210	54748	58522	48923	47559	**9.86**
**18**	45501	45128	43791	38180	37522	39640	**8.64**
**19**	95862	90839	80302	89970	81529	82902	**7.16**
**20**	48139	45484	49634	54920	49951	46215	**6.89**
**21**	66540	61216	66593	72142	63396	58104	**7.57**
**22**	264906	258907	242961	257032	261215	229588	**5.34**
**23**	463791	478726	440571	424254	413158	384744	**7.90**
**24**	83408	87679	83971	69304	73230	81171	**8.82**
**25**	224992	265331	243377	214815	213779	210528	**9.41**
**26**	173813	168834	168779	175495	169553	152827	**4.78**
**27**	39832	41497	39717	36842	35230	36354	**6.39**
**28**	74563	66113	63642	65274	69355	59849	**7.59**
**29**	85232	97810	82928	89290	83849	75519	**8.64**
**30**	80964	99180	89013	102463	89680	85386	**9.00**
**31**	102342	114353	92730	107049	96557	99264	**7.61**
**32**	60989	63334	66258	51653	56466	54605	**9.46**
**33**	79899	66192	69702	79822	77368	82279	**8.48**
**34**	65529	53294	54247	53932	58105	62753	**8.86**
**35**	227111	208392	179093	196112	177953	192793	**9.47**
**36**	218058	199686	212650	211490	215285	197458	**4.06**
**37**	70486	75933	65959	78077	64144	62952	**9.09**
**38**	114457	107956	111691	109677	105507	101361	**4.27**

**39**	75651	65700	61499	62129	64988	62965	**8.00**
**40**	102052	80133	80948	85009	84076	79758	**9.92**
**41**	69107	58881	53933	62632	58955	54884	**9.31**
**42**	411605	422329	394895	353462	361797	331029	**9.47**
**43**	120180	105805	99977	111980	99202	94662	**8.96**
**44**	66853	66305	61555	60190	57986	56383	**6.97**
**45**	96631	89502	82462	81230	80221	72400	**9.97**
**46**	85226	82275	72950	78588	80378	75831	**5.59**
**47**	100962	100895	88662	112652	100774	103328	**7.57**
**48**	154399	164379	148269	134275	155468	140745	**7.27**
**49**	80993	89104	91230	80402	76416	72172	**8.93**
**50**	117344	102697	92300	93365	94113	95777	**9.67**
**51**	164188	157440	141997	143384	139622	142657	**6.79**
**52**	90482	82908	73950	76037	77192	72481	**8.55**
**53**	69249	60743	59527	60380	51971	56140	**9.63**
**54**	45941	43592	45566	38968	39442	37707	**8.60**
**55**	76077	63315	59754	62891	66856	59399	**9.57**
**56**	71709	60612	58076	61631	56053	55326	**9.89**
**57**	23020	22623	21272	19234	18666	18647	**9.67**
**58**	27676	26817	27268	23219	27643	24851	**6.92**
**59**	32020	26622	25921	27792	25111	28739	**8.96**
**60**	9862	9099	9170	7853	7933	7882	**9.94**
**61**	21919	20073	18446	19180	16624	18636	**9.24**
**62**	8670	9097	10411	9290	8661	9552	**7.05**
**63**	5858	5655	5450	5016	5258	5587	**5.48**

Table S4. Repeatability investigation of Eupolyphaga steleophaga enzymolysis polypeptides retention time

**Peak No.**	**Repeated injection times**						**RSD%**
	1	2	3	4	5	6	

**1**	2.89	2.89	2.90	2.90	2.89	2.89	**0.12**
**2**	3.12	3.12	3.12	3.11	3.12	3.12	**0.18**
**3**	3.38	3.38	3.38	3.37	3.38	3.38	**0.09**
**4**	3.54	3.54	3.54	3.53	3.53	3.53	**0.20**
**5**	3.70	3.70	3.71	3.70	3.70	3.70	**0.08**
**6**	3.78	3.78	3.78	3.78	3.78	3.78	**0.02**
**7**	4.00	4.00	4.00	3.99	4.00	4.00	**0.16**
**8**	4.14	4.14	4.13	4.13	4.13	4.14	**0.08**
**9**	4.34	4.34	4.34	4.31	4.32	4.34	**0.33**
**10**	4.45	4.45	4.45	4.45	4.45	4.46	**0.01**
**11**	4.54	4.54	4.54	4.54	4.54	4.54	**0.01**
**12**	4.67	4.67	4.67	4.66	4.67	4.67	**0.07**
**13**	4.85	4.83	4.84	4.84	4.84	4.84	**0.10**
**14**	4.90	4.90	4.90	4.89	4.89	4.89	**0.08**
**15**	4.99	4.98	4.99	4.98	4.98	4.98	**0.07**
**16**	5.14	5.14	5.14	5.14	5.14	5.14	**0.01**
**17**	5.28	5.28	5.28	5.28	5.28	5.28	**0.06**
**18**	5.39	5.39	5.39	5.39	5.39	5.39	**0.01**
**19**	5.53	5.53	5.53	5.52	5.52	5.52	**0.10**
**20**	5.84	5.83	5.83	5.82	5.83	5.83	**0.09**
**21**	5.98	5.98	5.98	5.97	5.97	5.98	**0.06**
**22**	6.07	6.06	6.06	6.05	6.05	6.05	**0.09**
**23**	6.20	6.18	6.19	6.18	6.19	6.19	**0.09**
**24**	6.33	6.32	6.32	6.32	6.32	6.32	**0.05**
**25**	6.44	6.44	6.43	6.43	6.43	6.43	**0.05**
**26**	6.51	6.50	6.50	6.51	6.50	6.51	**0.02**
**27**	6.67	6.67	6.66	6.67	6.66	6.67	**0.06**
**28**	6.75	6.75	6.74	6.75	6.74	6.75	**0.06**
**29**	6.88	6.89	6.89	6.88	6.88	6.88	**0.05**

**30**	7.02	7.03	7.02	7.02	7.02	7.02	**0.03**
**31**	7.11	7.11	7.11	7.11	7.11	7.11	**0.05**
**32**	7.30	7.29	7.29	7.29	7.29	7.30	**0.04**
**33**	7.61	7.60	7.61	7.60	7.61	7.60	**0.05**
**34**	7.73	7.73	7.73	7.73	7.73	7.74	**0.05**
**35**	7.80	7.80	7.80	7.80	7.80	7.80	**0.04**
**36**	7.91	7.91	7.91	7.91	7.91	7.92	**0.03**
**37**	8.04	8.03	8.04	8.03	8.03	8.04	**0.05**
**38**	8.15	8.14	8.15	8.15	8.14	8.15	**0.08**
**39**	8.31	8.31	8.31	8.31	8.31	8.31	**0.03**
**40**	8.42	8.38	8.38	8.39	8.38	8.39	**0.15**
**41**	8.47	8.46	8.46	8.46	8.46	8.45	**0.05**
**42**	8.54	8.53	8.53	8.53	8.53	8.53	**0.04**
**43**	8.65	8.66	8.66	8.66	8.66	8.66	**0.04**
**44**	8.93	8.91	8.91	8.91	8.91	8.91	**0.08**
**45**	9.03	9.02	9.02	9.03	9.02	9.02	**0.06**
**46**	9.11	9.11	9.11	9.11	9.11	9.12	**0.04**
**47**	9.26	9.25	9.26	9.25	9.25	9.26	**0.04**
**48**	9.40	9.41	9.41	9.41	9.41	9.40	**0.04**
**49**	9.68	9.68	9.68	9.68	9.68	9.68	**0.02**
**50**	9.80	9.80	9.80	9.81	9.80	9.81	**0.02**
**51**	10.06	10.05	10.04	10.05	10.05	10.05	**0.05**
**52**	10.17	10.17	10.17	10.17	10.17	10.17	**0.03**
**53**	10.33	10.33	10.33	10.34	10.33	10.34	**0.02**
**54**	10.48	10.48	10.47	10.47	10.47	10.47	**0.03**
**55**	10.59	10.56	10.57	10.56	10.57	10.57	**0.09**
**56**	10.71	10.71	10.71	10.71	10.71	10.73	**0.07**
**57**	10.99	10.99	10.99	10.99	10.99	10.99	**0.02**
**58**	11.28	11.28	11.29	11.28	11.28	11.27	**0.05**

**59**	11.55	11.55	11.55	11.55	11.55	11.55	**0.02**
**60**	11.76	11.75	11.75	11.75	11.75	11.75	**0.05**
**61**	12.09	12.10	12.09	12.09	12.08	12.10	**0.04**
**62**	12.19	12.20	12.19	12.18	12.18	12.19	**0.08**
**63**	12.36	12.35	12.35	12.35	12.35	12.35	**0.03**

Table S5. Repeatability investigation of Eupolyphaga steleophaga enzymolysis polypeptides peak area

**Peak No.**	**Repeated injection times**						**RSD%**
	1	2	3	4	5	6	
**1**	15769	14811	15715	15109	13772	13150	**7.20**
**2**	4954	4235	4591	5093	4832	4916	**6.50**
**3**	48774	49558	49221	48892	41442	41884	**8.27**
**4**	29927	31385	29999	28285	26860	26532	**6.68**
**5**	39356	33651	31767	37450	33011	33952	**8.33**
**6**	87318	87870	87896	86823	84290	79976	**3.62**
**7**	12313	12737	12431	12673	12020	12041	**2.47**
**8**	14582	12204	13601	12579	11671	11377	**9.61**
**9**	41898	50782	47493	46724	48645	43107	**7.25**
**10**	42366	45589	41821	43680	43029	39612	**4.67**
**11**	34751	34531	32947	35369	35987	35461	**3.06**
**12**	82436	77325	79161	83112	79460	81436	**2.74**
**13**	9244	8914	9514	11320	10130	9862	**8.62**
**14**	55770	54725	62718	52784	52868	65709	**9.50**
**15**	53217	55666	53678	53004	49020	45909	**6.93**
**16**	15630	18237	16145	17125	15951	16305	**5.79**
**17**	59859	59210	54748	58522	48923	47559	**9.86**
**18**	45501	45128	43791	38180	37522	39640	**8.64**
**19**	95862	90839	80302	89970	81529	82902	**7.16**
**20**	48139	45484	49634	54920	49951	46215	**6.89**

**21**	66540	61216	66593	72142	63396	58104	**7.57**
**22**	264906	258907	242961	257032	261215	229588	**5.34**
**23**	463791	478726	440571	424254	413158	384744	**7.90**
**24**	83408	87679	83971	69304	73230	81171	**8.82**
**25**	224992	265331	243377	214815	213779	210528	**9.41**
**26**	173813	168834	168779	175495	169553	152827	**4.78**
**27**	39832	41497	39717	36842	35230	36354	**6.39**
**28**	74563	66113	63642	65274	69355	59849	**7.59**
**29**	75232	95810	82928	89290	83849	75519	**9.51**
**30**	80964	99180	79013	99463	89680	85386	**9.94**
**31**	102342	114353	92730	107049	96557	99264	**7.61**
**32**	60989	63334	66258	51653	56466	54605	**9.46**
**33**	79899	66192	69702	79822	77368	82279	**8.48**
**34**	65529	53294	54247	53932	58105	62753	**8.86**
**35**	217111	208392	179093	196112	177953	172793	**9.46**
**36**	218058	199686	212650	211490	215285	197458	**4.06**
**37**	70486	75933	65959	80077	64144	62952	**9.85**
**38**	114457	107956	111691	109677	105507	101361	**4.27**
**39**	75651	65700	61499	62129	56988	62965	**9.82**
**40**	102052	80133	80948	85009	84076	79758	**9.92**
**41**	69107	58881	53933	62632	58955	54884	**9.31**
**42**	411605	422329	394895	353462	361797	331029	**9.47**
**43**	120180	105805	99977	111980	99202	94662	**8.96**
**44**	66853	66305	61555	60190	57986	56383	**6.97**
**45**	96631	89502	82462	81230	80221	72400	**9.97**
**46**	85226	82275	72950	78588	80378	75831	**5.59**
**47**	100962	100895	88662	112652	100774	103328	**7.57**
**48**	154399	164379	148269	134275	155468	140745	**7.27**
**49**	80993	89104	91230	80402	76416	72172	**8.93**

**50**	117344	102697	92300	93365	94113	95777	**9.67**
**51**	164188	157440	141997	143384	139622	142657	**6.79**
**52**	90482	82908	73950	76037	77192	72481	**8.55**
**53**	69249	60743	59527	60380	51971	56140	**9.63**
**54**	45941	43592	45566	38968	39442	37707	**8.60**
**55**	76377	63315	59754	62891	66856	59399	**9.73**
**56**	71709	60612	58076	61631	56053	55326	**9.89**
**57**	23020	22623	21272	19234	18666	18647	**9.67**
**58**	27676	26817	27268	23219	27643	24851	**6.92**
**59**	32020	26622	25921	27792	25111	28739	**8.96**
**60**	9862	9099	9170	7853	7933	7882	**9.94**
**61**	21919	20073	18446	19180	16624	18636	**9.24**
**62**	8670	9097	10411	9290	8661	9552	**7.05**
**63**	5858	5655	5450	5016	5258	5587	**5.48**

Table S6. Stability investigation of Eupolyphaga steleophaga enzymolysis polypeptides retention time

**Peak No.**	**Repeated injection times**							**RSD%**
	0	2	4	6	8	10	12	
**1**	2.89	2.89	2.90	2.90	2.89	2.89	2.89	**0.12**
**2**	3.12	3.12	3.12	3.12	3.12	3.12	3.12	**0.12**
**3**	3.38	3.38	3.38	3.38	3.37	3.38	3.37	**0.10**
**4**	3.54	3.53	3.54	3.53	3.54	3.54	3.54	**0.17**
**5**	3.70	3.70	3.70	3.70	3.70	3.70	3.70	**0.01**
**6**	3.78	3.78	3.79	3.78	3.79	3.78	3.78	**0.09**
**7**	4.00	4.00	4.00	4.00	4.00	4.00	4.00	**0.09**
**8**	4.14	4.13	4.13	4.13	4.14	4.13	4.14	**0.08**
**9**	4.34	4.32	4.32	4.32	4.33	4.31	4.33	**0.26**
**10**	4.45	4.45	4.46	4.46	4.45	4.45	4.46	**0.08**

**11**	4.54	4.54	4.55	4.55	4.55	4.54	4.54	**0.09**
**12**	4.67	4.67	4.67	4.67	4.67	4.67	4.67	**0.01**
**13**	4.85	4.84	4.85	4.85	4.84	4.84	4.84	**0.07**
**14**	4.90	4.89	4.90	4.90	4.90	4.90	4.89	**0.07**
**15**	4.99	4.98	4.99	4.98	4.98	4.99	4.98	**0.08**
**16**	5.14	5.14	5.15	5.14	5.15	5.14	5.14	**0.07**
**17**	5.28	5.28	5.28	5.28	5.28	5.28	5.28	**0.07**
**18**	5.39	5.39	5.40	5.39	5.39	5.39	5.39	**0.05**
**19**	5.53	5.52	5.53	5.53	5.53	5.53	5.52	**0.06**
**20**	5.84	5.83	5.84	5.83	5.83	5.84	5.84	**0.07**
**21**	5.98	5.97	5.99	5.98	5.98	5.98	5.98	**0.08**
**22**	6.07	6.05	6.06	6.07	6.06	6.06	6.06	**0.09**
**23**	6.20	6.19	6.20	6.19	6.19	6.20	6.19	**0.07**
**24**	6.33	6.32	6.33	6.33	6.33	6.33	6.32	**0.06**
**25**	6.44	6.43	6.43	6.44	6.43	6.43	6.43	**0.05**
**26**	6.51	6.50	6.50	6.50	6.50	6.51	6.50	**0.01**
**27**	6.67	6.66	6.67	6.67	6.67	6.67	6.67	**0.04**
**28**	6.75	6.74	6.74	6.75	6.75	6.75	6.75	**0.06**
**29**	6.88	6.88	6.89	6.89	6.88	6.88	6.89	**0.06**
**30**	7.02	7.02	7.02	7.02	7.02	7.02	7.03	**0.04**
**31**	7.11	7.11	7.11	7.11	7.11	7.10	7.11	**0.07**
**32**	7.30	7.29	7.30	7.30	7.29	7.30	7.30	**0.05**
**33**	7.61	7.61	7.61	7.61	7.61	7.61	7.61	**0.01**
**34**	7.73	7.73	7.74	7.74	7.73	7.73	7.73	**0.06**
**35**	7.80	7.80	7.80	7.80	7.80	7.80	7.81	**0.04**
**36**	7.91	7.91	7.91	7.91	7.91	7.91	7.92	**0.01**
**37**	8.04	8.03	8.04	8.04	8.04	8.04	8.04	**0.04**
**38**	8.15	8.14	8.15	8.15	8.15	8.15	8.15	**0.05**
**39**	8.31	8.31	8.31	8.31	8.31	8.31	8.31	**0.05**

**40**	8.42	8.38	8.42	8.41	8.38	8.39	8.39	**0.19**
**41**	8.47	8.46	8.47	8.47	8.47	8.46	8.47	**0.04**
**42**	8.54	8.53	8.54	8.54	8.53	8.54	8.54	**0.05**
**43**	8.65	8.66	8.66	8.66	8.66	8.66	8.66	**0.04**
**44**	8.93	8.91	8.91	8.91	8.91	8.91	8.91	**0.07**
**45**	9.03	9.02	9.02	9.03	9.02	9.02	9.03	**0.06**
**46**	9.11	9.11	9.12	9.11	9.11	9.12	9.11	**0.05**
**47**	9.26	9.25	9.26	9.26	9.26	9.26	9.26	**0.04**
**48**	9.40	9.41	9.42	9.41	9.40	9.41	9.41	**0.05**
**49**	9.68	9.68	9.69	9.69	9.68	9.68	9.68	**0.06**
**50**	9.80	9.80	9.80	9.80	9.80	9.81	9.81	**0.01**
**51**	10.06	10.05	10.05	10.06	10.05	10.04	10.05	**0.05**
**52**	10.17	10.17	10.17	10.17	10.17	10.17	10.17	**0.04**
**53**	10.33	10.33	10.33	10.33	10.33	10.34	10.34	**0.01**
**54**	10.48	10.47	10.47	10.48	10.47	10.47	10.48	**0.04**
**55**	10.59	10.57	10.57	10.57	10.56	10.56	10.57	**0.09**
**56**	10.71	10.71	10.70	10.71	10.70	10.71	10.71	**0.03**
**57**	10.99	10.99	10.99	10.99	10.99	10.99	10.99	**0.03**
**58**	11.28	11.28	11.28	11.29	11.28	11.28	11.28	**0.03**
**59**	11.55	11.55	11.56	11.56	11.55	11.55	11.56	**0.03**
**60**	11.76	11.75	11.76	11.76	11.75	11.75	11.77	**0.06**
**61**	12.09	12.08	12.10	12.09	12.09	12.09	12.10	**0.04**
**62**	12.19	12.18	12.19	12.19	12.18	12.17	12.19	**0.07**
**63**	12.36	12.35	12.35	12.35	12.35	12.35	12.35	**0.03**

Table S7. Stability investigation of Eupolyphaga steleophaga enzymolysis polypeptides peak area

**Peak No.**	**Repeated injection times**							**RSD%**
	0	2	4	5	8	10	12	
**1**	15769	13772	15638	14358	14069	12608	12965	**8.55**

**2**	4954	4832	5486	5891	4883	5003	5673	**8.17**
**3**	48774	41442	52039	48340	46467	45960	42039	**8.10**
**4**	29927	26860	30151	30321	29339	29302	27830	**4.44**
**5**	39356	33011	36399	34365	33846	34675	32647	**6.64**
**6**	87318	84290	92527	84330	84986	80633	80149	**4.94**
**7**	12313	12020	11468	12554	15238	12824	12726	**9.40**
**8**	14582	11671	11212	12540	13562	11485	12055	**9.85**
**9**	41898	48645	42347	45112	46878	38833	42205	**7.68**
**10**	42366	43029	41723	43556	42498	37750	37261	**6.24**
**11**	34751	35987	36589	34736	34487	28889	28546	**9.89**
**12**	82436	79460	84066	84844	78792	73441	71725	**6.42**
**13**	9244	10130	9384	11890	9736	10031	8860	**9.96**
**14**	55770	52868	68446	55376	64477	58718	57684	**9.33**
**15**	53217	49020	54061	57286	55204	48593	43553	**9.18**
**16**	15630	15951	15334	18966	17402	17417	16215	**7.71**
**17**	61859	48923	53947	59577	58514	50331	48991	**9.90**
**18**	45501	37522	37631	42355	43170	35972	37680	**9.09**
**19**	95862	81529	80691	94909	91782	77195	76625	**9.81**
**20**	48139	49951	56932	54368	43505	44946	47817	**9.80**
**21**	66540	63396	67745	71694	55718	63319	59890	**8.21**
**22**	264906	261215	269007	243849	241702	236284	231510	**6.00**
**23**	463791	413158	471910	429537	425455	383016	357064	**9.76**
**24**	83408	73230	90847	88452	76262	75594	68695	**9.83**
**25**	224992	213779	248331	207978	229932	215245	178393	**9.95**
**26**	173813	169553	168721	173738	166044	140726	141717	**8.94**
**27**	38832	35230	33609	30377	31162	31817	30813	**9.20**
**28**	74563	69355	74044	64993	61446	71723	77532	**8.06**
**29**	75232	83849	91356	89916	83220	71421	73488	**9.81**
**30**	80964	89680	96161	96450	92099	89991	84390	**6.37**

**31**	102342	96557	106403	110016	98338	99782	91227	**6.22**
**32**	60989	56466	62486	56841	52458	52960	47476	**9.32**
**33**	79899	77368	75234	82769	79378	72558	77198	**4.27**
**34**	65529	58105	65598	64708	61723	54765	54515	**8.09**
**35**	227111	177953	214754	194118	193997	188798	181885	**9.03**
**36**	218058	215285	215608	202022	204548	191472	186642	**6.04**
**37**	70486	64144	73920	58487	63863	70480	62900	**8.17**
**38**	114457	105507	99715	96535	99069	97398	85390	**6.67**
**39**	74651	54988	64899	64411	59841	59425	63416	**9.78**
**40**	101952	84076	84132	85176	83184	77103	77457	**9.77**
**41**	68107	58955	63891	59489	57379	53732	50705	**9.95**
**42**	411605	361797	398284	372278	369061	346539	347214	**6.61**
**43**	120180	99202	112370	102620	98831	99518	97286	**8.29**
**44**	63853	57986	63728	55274	61550	55437	47719	**9.95**
**45**	96631	80221	90635	88535	82780	79709	76583	**8.40**
**46**	85226	80378	90183	86737	82083	76875	70883	**7.92**
**47**	100962	100774	105310	102546	99004	100150	87700	**5.61**
**48**	154399	155468	159044	149695	150352	153049	131900	**5.85**
**49**	80993	76416	75354	72858	70453	69344	65448	**7.03**
**50**	113344	94113	115638	102360	93701	95690	90510	**9.98**
**51**	164188	139622	158464	151714	138594	144899	122933	**9.49**
**52**	90482	77192	85010	79019	76763	73902	66776	**9.73**
**53**	68249	51971	61964	61460	55910	55645	53637	**9.81**
**54**	45941	39442	44351	39146	41114	39685	34051	**9.56**
**55**	78377	66856	66831	68609	67843	67602	74072	**6.36**
**56**	70709	56053	69840	60937	60843	59898	55385	**9.85**
**57**	23020	18666	20578	19285	19196	17746	18180	**9.16**
**58**	27676	27643	25810	24626	23662	22419	21506	**9.78**
**59**	32020	25111	26134	27403	25806	25318	24598	**9.54**

**60**	9962	7933	7992	9225	8571	8927	9064	**8.12**
**61**	21919	17624	20571	17336	19401	17916	17183	**9.72**
**62**	8670	8661	8723	7808	8213	8761	7421	**6.35**
**63**	5858	5258	5983	5351	6252	5351	5294	**7.15**

Table S8. Retention time and RSDs of 10 batches Eupolyphaga steleophaga enzymolysis polypeptides

Peak	Batches										RSD
No.	1	2	3	4	5	6	7	8	9	10	%
**1**	3.02	3.02	3.01	3.02	3.02	3.02	3.02	3.02	3.01	3.01	**0.16**
**2**	3.23	3.22	3.22	3.23	3.22	3.23	3.22	3.23	3.22	3.22	**0.16**
**3**	3.49	3.49	3.49	3.49	3.49	3.49	3.49	3.49	3.49	3.48	**0.09**
**4**	3.64	3.64	3.65	3.65	3.64	3.64	3.65	3.64	3.64	3.64	**0.13**
**5**	3.80	3.81	3.80	3.80	3.80	3.80	3.81	3.80	3.80	3.80	**0.11**
**6**	3.88	3.88	3.88	3.89	3.89	3.89	3.88	3.88	3.88	3.88	**0.12**
**7**	4.10	4.10	4.11	4.11	4.10	4.10	4.10	4.11	4.10	4.10	**0.12**
**8**	4.26	4.25	4.26	4.26	4.26	4.26	4.25	4.26	4.26	4.25	**0.11**
**9**	4.44	4.44	4.43	4.44	4.44	4.43	4.44	4.44	4.44	4.44	**0.10**
**10**	4.58	4.59	4.59	4.58	4.58	4.58	4.58	4.58	4.59	4.58	**0.11**
**11**	4.69	4.68	4.69	4.68	4.69	4.69	4.68	4.68	4.68	4.68	**0.11**
**12**	4.77	4.77	4.78	4.77	4.77	4.77	4.78	4.77	4.77	4.77	**0.09**
**13**	4.96	4.96	4.96	4.95	4.95	4.96	4.95	4.95	4.96	4.95	**0.11**
**14**	5.04	5.05	5.04	5.04	5.04	5.05	5.04	5.04	5.04	5.04	**0.08**
**15**	5.11	5.11	5.10	5.10	5.11	5.11	5.11	5.11	5.11	5.10	**0.09**
**16**	5.28	5.28	5.27	5.28	5.28	5.28	5.27	5.28	5.28	5.29	**0.11**
**17**	5.41	5.41	5.41	5.42	5.42	5.42	5.42	5.42	5.41	5.41	**0.10**
**18**	5.51	5.51	5.51	5.50	5.51	5.51	5.50	5.51	5.51	5.51	**0.08**
**19**	5.67	5.66	5.66	5.67	5.67	5.67	5.67	5.67	5.67	5.66	**0.09**
**20**	6.04	6.04	6.05	6.04	6.05	6.04	6.04	6.04	6.04	6.04	**0.07**
**21**	6.11	6.11	6.10	6.10	6.10	6.11	6.10	6.11	6.10	6.10	**0.08**
**22**	6.20	6.20	6.19	6.20	6.20	6.20	6.20	6.20	6.20	6.20	**0.05**
**23**	6.31	6.29	6.31	6.31	6.31	6.31	6.31	6.31	6.31	6.29	**0.13**
**24**	6.43	6.43	6.43	6.43	6.44	6.44	6.44	6.43	6.43	6.43	**0.08**
**25**	6.54	6.54	6.54	6.55	6.54	6.54	6.55	6.54	6.54	6.54	**0.06**
**26**	6.60	6.59	6.59	6.59	6.59	6.59	6.60	6.60	6.60	6.60	**0.08**

**27**	6.77	6.78	6.78	6.78	6.77	6.77	6.77	6.78	6.77	6.77	**0.08**
**28**	6.84	6.83	6.83	6.84	6.84	6.84	6.84	6.84	6.84	6.83	**0.07**
**29**	6.99	6.99	6.99	6.98	6.98	6.99	6.99	6.98	6.99	6.99	**0.07**
**30**	7.10	7.10	7.10	7.10	7.11	7.11	7.11	7.11	7.11	7.11	**0.07**
**31**	7.21	7.21	7.21	7.22	7.22	7.22	7.22	7.22	7.21	7.21	**0.07**
**32**	7.37	7.38	7.38	7.38	7.37	7.37	7.38	7.38	7.38	7.37	**0.07**
**33**	7.68	7.68	7.68	7.68	7.68	7.68	7.68	7.68	7.68	7.68	**0.00**
**34**	7.82	7.82	7.82	7.82	7.82	7.83	7.83	7.82	7.82	7.82	**0.05**
**35**	7.90	7.90	7.90	7.90	7.90	7.90	7.90	7.90	7.90	7.90	**0.00**
**36**	8.01	8.01	8.02	8.01	8.02	8.01	8.01	8.02	8.02	8.01	**0.06**
**37**	8.12	8.12	8.12	8.12	8.12	8.12	8.12	8.12	8.11	8.12	**0.04**
**38**	8.24	8.24	8.24	8.24	8.25	8.25	8.25	8.25	8.25	8.25	**0.06**
**39**	8.41	8.41	8.41	8.41	8.41	8.42	8.41	8.41	8.41	8.41	**0.04**
**40**	8.54	8.54	8.54	8.54	8.54	8.54	8.54	8.54	8.53	8.53	**0.05**
**41**	8.57	8.57	8.57	8.57	8.58	8.58	8.58	8.58	8.58	8.57	**0.06**
**42**	8.64	8.64	8.65	8.65	8.64	8.64	8.65	8.65	8.65	8.64	**0.06**
**43**	8.76	8.76	8.77	8.77	8.77	8.76	8.76	8.76	8.77	8.76	**0.06**
**44**	9.01	9.01	9.02	9.01	9.01	9.01	9.01	9.01	9.01	9.01	**0.04**
**45**	9.14	9.13	9.14	9.14	9.14	9.14	9.14	9.13	9.13	9.13	**0.06**
**46**	9.21	9.21	9.21	9.20	9.20	9.20	9.20	9.21	9.21	9.21	**0.06**
**47**	9.36	9.36	9.36	9.36	9.34	9.34	9.34	9.34	9.34	9.34	**0.11**
**48**	9.51	9.51	9.51	9.52	9.52	9.52	9.52	9.52	9.52	9.51	**0.05**
**49**	9.78	9.77	9.78	9.78	9.78	9.78	9.78	9.77	9.78	9.78	**0.04**
**50**	9.90	9.90	9.90	9.90	9.90	9.90	9.90	9.90	9.90	9.90	**0.00**
**51**	10.16	10.16	10.16	10.16	10.16	10.16	10.16	10.16	10.16	10.16	**0.00**
**52**	10.27	10.27	10.28	10.28	10.28	10.27	10.27	10.28	10.27	10.27	**0.05**
**53**	10.43	10.42	10.43	10.42	10.42	10.43	10.43	10.42	10.42	10.42	**0.05**
**54**	10.58	10.58	10.58	10.58	10.58	10.58	10.58	10.58	10.57	10.58	**0.03**
**55**	10.65	10.64	10.64	10.65	10.65	10.65	10.65	10.65	10.65	10.65	**0.04**

**56**	10.79	10.79	10.80	10.79	10.79	10.80	10.79	10.79	10.79	10.79	**0.04**
**57**	11.07	11.07	11.07	11.08	11.07	11.07	11.08	11.07	11.08	11.07	**0.04**
**58**	11.40	11.40	11.40	11.41	11.40	11.40	11.40	11.41	11.40	11.40	**0.04**
**59**	11.67	11.67	11.67	11.67	11.67	11.67	11.67	11.67	11.66	11.67	**0.03**
**60**	11.85	11.84	11.84	11.84	11.85	11.85	11.85	11.84	11.84	11.84	**0.04**
**61**	12.22	12.22	12.22	12.22	12.22	12.22	12.22	12.21	12.21	12.21	**0.04**
**62**	12.27	12.27	12.27	12.28	12.28	12.27	12.27	12.27	12.28	12.28	**0.04**
**63**	12.44	12.44	12.44	12.44	12.44	12.44	12.45	12.45	12.44	12.44	**0.03**

Table S9. Peak area and RSDs of 10 batches Eupolyphaga steleophaga enzymolysis polypeptides

**Peak No.**	**Batches**										**RSD%**
	**1**	**2**	**3**	**4**	**5**	**6**	**7**	**8**	**9**	**10**	
**1**	8260	8223	8316	8222	8791	8388	8199	8414	6521	6520	**9.90**
**2**	3136	2992	3114	2941	2782	2663	2790	2888	3395	3352	**8.09**
**3**	6936	6756	7135	7158	5618	5900	5669	5767	6175	5931	**9.90**
**4**	1330	1225	1433	1366	1121	1316	1334	1139	1179	1129	**8.93**
**5**	51876	54689	51279	51219	56518	52210	51926	55649	52322	52191	**3.59**
**6**	516	439	563	512	534	511	460	488	615	498	**9.74**
**7**	7103	7101	7199	7170	7120	7189	7112	7110	7110	7190	**0.57**
**8**	2425	2405	2378	2336	2313	2243	2223	2299	2093	2091	**5.19**
**9**	20523	22226	19891	20528	22637	22846	22166	22915	23003	22953	**5.42**
**10**	12338	11916	11251	11151	12009	11813	11649	11722	11363	11111	**3.49**
**11**	6381	6326	6866	7267	7476	7458	7309	7453	5956	5928	**9.31**
**12**	17764	17595	17121	17448	20281	17323	17232	18097	14684	14378	**9.72**
**13**	6214	6099	6025	5803	5758	5615	5581	5690	5240	5158	**6.02**
**14**	8146	8682	8760	8256	8845	8899	10601	10591	8805	8680	**9.56**
**15**	9141	9130	8930	8847	10090	10043	9882	9941	7964	7653	**9.37**
**16**	4089	3986	3793	4043	4244	4141	4218	4261	3463	4126	**6.06**
**17**	15711	15909	15520	15790	18537	18559	18161	18496	19724	18830	**9.10**

**18**	6284	5970	5976	6116	7510	7569	7072	7510	6583	6886	**9.63**
**19**	27711	26873	26283	27050	28622	28370	27796	28530	26116	26226	**3.58**
**20**	1149	1184	1163	1124	996	933	950	989	1067	1052	**8.63**
**21**	27879	23430	26904	27651	24934	26043	25902	24524	30087	29045	**7.80**
**22**	82101	79375	79209	78032	77791	78230	75899	78303	79249	75698	**2.33**
**23**	71718	68740	68045	71061	73629	73043	73746	73617	64051	62430	**5.86**
**24**	19244	17664	18253	17240	19743	20340	19840	19871	19221	18322	**5.50**
**25**	1204	1093	1031	1102	1239	1040	1367	1177	1201	1016	**9.68**
**26**	1231	1622	1360	1398	1456	1477	1412	1653	1464	1289	**9.17**
**27**	5855	5617	5104	5947	5109	5368	5262	6660	5367	5558	**8.48**
**28**	11935	11705	11661	11535	12808	12782	12661	12744	10777	10851	**6.52**
**29**	31431	30957	31236	30547	31204	30572	30415	31221	34025	33949	**4.20**
**30**	5874	5774	6071	5243	5739	5190	5454	5228	5590	5655	**5.35**
**31**	428	619	584	547	501	530	557	527	599	554	**9.93**
**32**	8545	8417	8173	8357	8248	8933	8779	9369	8899	7851	**5.18**
**33**	6994	6723	7016	6815	7921	6143	6103	7611	6282	5876	**9.90**
**34**	12634	12680	11865	12575	14380	13301	13725	12189	10684	10652	**9.62**
**35**	49566	47947	48721	42521	44761	51433	50493	49699	45659	41918	**7.11**
**36**	5792	5247	5707	5744	4584	5085	4714	4655	5792	5919	**9.99**
**37**	29906	28209	29266	29055	25741	26295	26021	26086	33332	33404	**10.00**
**38**	38221	36339	37736	38196	37272	40693	41098	38827	34691	34587	**5.79**
**39**	7423	7137	7312	7079	8460	9120	9016	8426	8195	8921	**9.94**
**40**	4995	4143	5013	4335	5007	4737	4386	4714	4913	5217	**7.41**
**41**	28794	28397	24131	28312	29373	30664	29471	29665	22332	31222	**10.00**
**42**	93189	91286	92037	93224	102141	105251	105454	103186	84760	84898	**8.30**
**43**	9341	9165	9102	9314	9894	9155	9137	9642	9164	9176	**2.80**
**44**	27854	30715	27410	27331	28465	28418	26366	26704	30608	32165	**6.74**
**45**	13800	13583	13656	13731	13521	14019	13920	13869	14043	11522	**5.45**
**46**	11745	11282	11237	11366	12647	11357	12504	12372	12162	9281	**8.42**

**47**	12149	12139	11116	12387	12326	12461	12459	13243	12516	13522	**5.20**
**48**	141162	145381	142231	150847	143477	158503	160094	157003	144573	138084	**5.34**
**49**	17313	16671	16689	16761	18374	17356	16992	21935	16563	15550	**9.99**
**50**	53408	55178	51990	51275	54154	57194	56330	56855	53451	66717	**7.85**
**51**	1118	1159	1131	1142	1519	1233	1262	1243	1131	1184	**9.87**
**52**	1153	1116	1198	1425	1291	1082	1166	1097	1197	1393	**9.91**
**53**	20809	20165	20496	20098	20122	21370	21326	21010	21301	20669	**2.45**
**54**	19238	18991	18623	18432	21661	20723	20697	20670	23603	23001	**8.73**
**55**	38731	37389	34538	36565	37915	37345	36848	35976	41183	39365	**4.94**
**56**	35580	34120	33916	33233	29008	28343	27835	28121	33849	33348	**9.50**
**57**	8256	8175	8147	8138	8282	8381	7808	7390	7539	7648	**4.39**
**58**	7523	7524	7519	7393	8626	7714	7428	8180	7818	7715	**4.99**
**59**	6225	6142	5917	5867	6405	6502	6503	6447	5916	6121	**4.05**
**60**	4841	4947	5032	5018	5440	5374	5222	5242	5342	5213	**3.84**
**61**	3119	3013	3010	3314	3513	3415	3212	3501	3088	2505	**9.48**
**62**	1477	1501	1498	1361	1321	1275	1273	1286	1147	1514	**9.24**
**63**	1404	1414	1325	1319	1129	1077	1220	1228	1224	1129	**9.34**
